# Extensive Cotransformation of Natural Variation into Chromosomes of Naturally Competent *Haemophilus influenzae*

**DOI:** 10.1534/g3.113.009597

**Published:** 2014-02-25

**Authors:** Joshua Chang Mell, Jae Yun Lee, Marlo Firme, Sunita Sinha, Rosemary J. Redfield

**Affiliations:** * Department of Zoology, University of British Columbia, Vancouver, BC V6T 1Z3, Canada; † Genome Sciences and Technology Graduate Program, University of British Columbia, Vancouver, BC V6T 1Z3, Canada; ‡ Department of Pharmacy Sciences, University of British Columbia, Vancouver, BC V6T 1Z3, Canada

**Keywords:** recombination, bacteria, horizontal gene transfer, heteroduplex segregation, nearly isogenic lines

## Abstract

Naturally competent bacterial species actively take up environmental DNA and can incorporate it into their chromosomes by homologous recombination. This can bring genetic variation from environmental DNA to recipient chromosomes, often in multiple long “donor” segments. Here, we report the results of genome sequencing 96 colonies of a laboratory *Haemophilus influenzae* strain, which had been experimentally transformed by DNA from a diverged clinical isolate. Donor segments averaged 6.9 kb (spanning several genes) and were clustered into recombination tracts of ~19.5 kb. Individual colonies had replaced from 0.1 to 3.2% of their chromosomes, and ~1/3 of all donor-specific single-nucleotide variants were present in at least one recombinant. We found that nucleotide divergence did not obviously limit the locations of recombination tracts, although there were small but significant reductions in divergence at recombination breakpoints. Although indels occasionally transformed as parts of longer recombination tracts, they were common at breakpoints, suggesting that indels typically block progression of strand exchange. Some colonies had recombination tracts in which variant positions contained mixtures of both donor and recipient alleles. These tracts were clustered around the origin of replication and were interpreted as the result of heteroduplex segregation in the original transformed cell. Finally, a pilot experiment demonstrated the utility of natural transformation for genetically dissecting natural phenotypic variation. We discuss our results in the context of the potential to merge experimental and population genetic approaches, giving a more holistic understanding of bacterial gene transfer.

Many bacterial species are naturally competent, able to take up DNA from their environment and incorporate it into their chromosome by homologous recombination (Chen and Dubnau 2004; Claverys *et al.* 2009; Johnsborg *et al.* 2007; Kruger and Stingl 2011; Thomas and Nielsen 2005). This natural transformation is the major pathway of genetic transfer between related competent lineages, and it has had a profound influence on bacterial evolution, transferring both allelic variation and whole loci between lineages, similar to the genetic shuffling generated during eukaryotic sexual reproduction (Moradigaravand and Engelstadter 2013; Vos and Didelot 2009).

Comparing DNA sequences from natural bacterial isolates has found that intraspecific recombination is often common, especially in species known to be naturally competent (Didelot and Maiden 2010; Feil *et al.* 2001; Feil and Spratt 2001; Perez-Losada *et al.* 2006). In pathogens these recombination events appear to have promoted adaptation to host defenses and antibiotic resistance (Croucher *et al.* 2011; Hanage *et al.* 2009). Interpreting this historical evidence, however, first requires disentangling the immediate consequences of recombination from the other evolutionary forces that act on recombinant genomes, such as mutation, selection, and population substructure (Feil and Spratt 2001). To do this, we need to first understand how the mechanism of natural transformation determines the distribution of donor genetic variation brought into recipient chromosomes.

In contrast to the recombination that repairs DNA double-stranded breaks (for example, that arising during DNA replication and meiotic recombination), the donor DNAs in natural transformation are transported into the cytoplasm as single strands with their 3′-end leading through a conserved membrane pore (Rec2/ComEC), whereas the complementary strands are simultaneously degraded. If translocated single-stranded DNA (ssDNA) has high sequence similarity to a segment of the competent cell’s chromosome, it can undergo recombination once coated with the strand exchange ATPase RecA; otherwise, translocated DNAs also are degraded. Thus transformational recombination occurs without the formation or resolution of Holliday junctions, since the substrates are ssDNA donors and dsDNA recipients (Claverys *et al.* 2009; Kruger and Stingl 2011; Maughan *et al.* 2008; Mortier-Barriere *et al.* 2007; Pifer and Smith 1985) (Figure 1).

**Figure 1:**
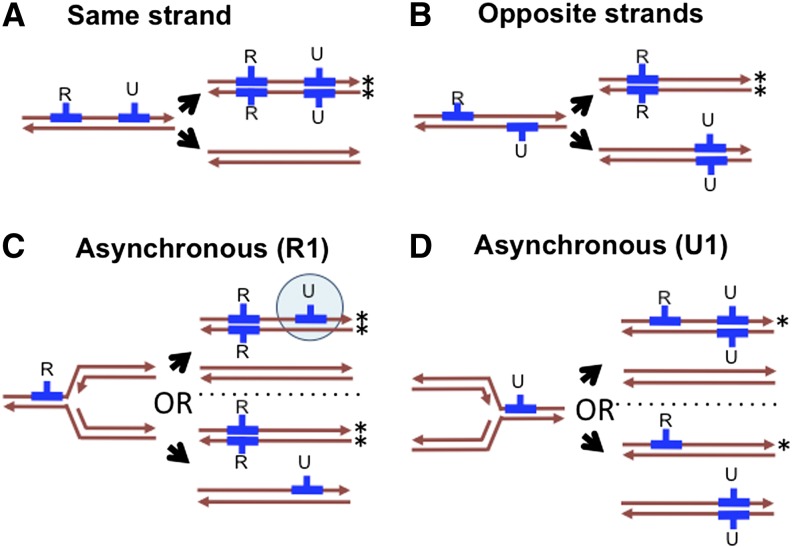
Model of transformation and possible outcomes of heteroduplex segregation for two independent donor DNAs (blue) in the absence of heteroduplex correction. One fragment carries a selectable antibiotic resistance allele (R); the other is unselected (U). Strands that go on to produce a resistant colony are indicated with an asterisk (*). (A) The U fragment replaces resident sequences on the same strand as the R fragment, leading to a resistant colony homogeneous for the R and U recombination tracts. (B) The U fragment replaces the opposite strand as the R fragments, leading to a resistant colony with only the R tract. (C) and (D) are as in (A), except that the two donor molecules transform asynchronously with respect to segregation. This could be either temporal (one fragment transformed before replication and the other after) or spatial (the replication fork had passed through one locus but not the other when the fragments transformed). (C) The R fragment transforms first, leading to a colony either heterogeneous for the U fragment (shown by the blue circle) or missing the U fragment, depending on the strands transformed. (D) The U fragment transforms first, leading to a resistant colony homogeneous for either both fragments or only the R fragment.

Unless mismatch repair intervenes, the resulting heteroduplex recombination products (genetically equivalent to gene conversion intermediates) will simply segregate upon DNA replication to give rise to two homoduplex daughter molecules, one carrying donor alleles and the other unchanged from the recipient. This heteroduplex segregation can cause single cells transformed by more than one donor DNA molecule to give rise to genetically heterogeneous daughter cells (Figure 1, A and B). Such heterogeneity would be even more frequent when independent DNAs transform recipient chromosomes asynchronously with respect to DNA replication, with doubly transformed chromosomes giving rise to potentially >2 distinct genotypes, depending on which strand of each donor molecule had been translocated into the cytoplasm (Figure 1, C and D).

Further complicating the picture, previous studies of natural transformation have shown that contiguous runs of donor-specific variation (“donor segments”) often are clustered into complex mosaic patterns, suggesting that long DNA molecules frequently are disrupted after uptake. This could be due to either endonucleases acting on DNA prior to strand exchange or heteroduplex correction mechanisms acting after recombination products are formed (Croucher *et al.* 2012; Lin *et al.* 2009; Mell *et al.* 2011).

Although recombination tracts are limited by the size distribution of donor DNA fragments, they can span many genes and typically include regions of both high and low nucleotide divergence between donor and recipient (Croucher *et al.* 2012; Lin *et al.* 2009; Mell *et al.* 2011). Further, structural variants (SVs; insertions, deletions, and other rearrangements) can cotransform with flanking markers as parts of longer recombination tracts, though they appear to transform less readily than single-nucleotide variants (SNVs) (Mell *et al.* 2011; Stuy and Walter 1981).

Previous experimental work has shown that naturally competent cells commonly take up and recombine more than one independent DNA molecule. This permits use of cotransformation frequencies to investigate properties of the competent cell population. Such analysis frequently finds unexpectedly high cotransformation frequencies of markers on independent molecules (“congression”), indicating that only a fraction of cells in a competent culture take up and recombine DNA (Goodgal and Herriott 1961; Mell *et al.* 2011; but see Erickson and Copeland 1973). Although congression suggests that competence may be an on/off cellular state, it could also reflect more continuous variation in the transformability of competent cells (Maamar *et al.* 2007).

Experimental studies of natural transformation have typically focused on the extent of recombination around selectable antibiotic resistance markers, but the advent of inexpensive DNA sequencing allows investigation of its genome-wide effects (Croucher *et al.* 2012; Mell *et al.* 2011; Power *et al.* 2012). Wild strains typically differ at thousands of positions, and recombinants can be comprehensively genotyped by genome sequencing, thereby identifying recombinant segments with high precision. Such recombinants also more closely mirror the most interesting natural conditions, where diverged strains in polyclonal populations can generate complex recombinants. Eventually, such work will contribute to a synthesis between experimental studies of recombination and evolutionary studies of its consequences.

Here we report work using *Haemophilus influenzae*, a model system for natural competence and an important member of the human microbiome. *H. influenzae* is primarily found as a commensal of the upper respiratory tract in children but is also often an agent of serious disease, especially in patients with respiratory conditions such as cystic fibrosis and chronic obstructive pulmonary disease (Agrawal and Murphy 2011; Dworkin *et al.* 2007; MacNeil *et al.* 2011). We previously reported the use of genome sequencing to identify all the genetic changes in four *Haemophilus influenzae* clones transformed by DNA from a divergent strain (Mell *et al.* 2011). The clones had replaced 1–3% of their chromosomes with donor DNA at independent locations, underlining the genetic consequences of transformation for single competent cells. However, the number of clones investigated was too small for statistical analysis of either transformation’s overall extent in individual cells or its distribution around the chromosome. The work here updates our comprehensive genotyping approach and extends genome-wide analysis to a larger set of 96 transformed chromosomes, thereby providing parameter estimates that will be useful for inferring historical recombination in natural populations. We also show that natural transformation offers a potentially powerful approach to mapping the underlying genetic causes of natural phenotypic variation in competent bacterial species.

## Materials and Methods

### Collection of transformed clones

*Haemophilus influenzae* strains are listed in Table 1 and detailed in Supporting Information, File S2. Culturing and manipulation of *H. influenzae* were performed via standard methods (Poje and Redfield 2003a). Competent cells of the Rd strain (KW20, RR722) (Fleischmann *et al.* 1995; Wilcox and Smith 1975) were prepared by transfer of exponentially growing cells to MIV starvation medium, as described (Poje and Redfield 2003b; Steinhart and Herriott 1968) and incubated with genomic DNA extracted from donor strains [~1 genome equivalent per cell, ~2 µg/10^9^ colony-forming units (CFUs) in 1 mL] at 37° for 30 min. Cultures were then treated with DNase I (1 µg/mL) for 10 min to degrade extracellular donor fragments, followed by 1:5 dilutions into the rich medium sBHI (Difco brain-heart infusion supplemented with 10μg/ml hemin and 2μg/ml NAD) and incubation at 37° for 80 min to allow expression of the Nal^R^ resistance allele. A late-log transformation was conducted in the same manner, except using a culture grown in sBHI that had reached stationary phase (OD_600_ = 1.2).

**Table 1:** Transformation experiments and clone isolation

		Cell Preparation*^a^*			
Donor DNA	Value	MIV-1	MIV-2	MIV-3	Late-log
RR666 donor DNA	Nov^R^/CFU	6.40 × 10^−4^	9.20 × 10^−4^	2.40 × 10^−3^	1.33 × 10^−5^
	Nal^R^/CFU	9.50 × 10^−4^	4.00 × 10^−3^	2.50 × 10^−3^	ND
	Congression*^b^*	14.5	8.7	7.0	ND
RR3131 donor DNA	Nov^R^/CFU	2.70 × 10^−4^	6.10 × 10^−4^	7.30 × 10^−4^	1.15 × 10^−5^
	Nal^R^/CFU	8.50 × 10^−4^	1.10 × 10^−3^	8.80 × 10^−4^	ND
	Congression*^b^*	9.6	8.2	6.2	ND
Strains collected and sequenced*^c^*	Nal^R^ (36)	RR4001-12	RR4033-44	RR4065-76	ND
	Nov^R^ (44)	RR4013-24	RR4045-56	RR4077-88	RR4117-24
	None (16)*^d^*	RR4025-26	ND	RR4101-10,13-16	ND

CFU, colony-forming unit; ND , no data.

a Three independent MIV cultures of the lab strain Rd (also known as KW20, strain RR722) were prepared and transformed with genomic DNA from either MAP7 (RR666) or a Nov^R^ Nal^R^ derivative of 86-028NP (RR3131) as donor [(DNA) ~1 genome/cell]. A fourth late-log culture (OD_600_ = 1.2) also was transformed. The transformation frequency of the donor Nov^R^ and Nal^R^ alleles were measured by standard plating assay.

b Congression was measured as the observed:expected ratio of Nov^R^Nal^R^ recombinants, or (Nov^R^Nal^R^/CFU) / (Nov^R^/CFU * Nal^R^/CFU).

c Isolated colonies were propagated from each transformation with RR3131 donor DNA for genome sequencing. The antibiotic used for selection is indicated in column 2, with the total number of isolated clones indicated in parentheses. Strain numbers are indicated for each selected type (Table S1). In addition to the strains listed here, the donor and recipient genomes were sequenced in parallel as controls (RR722, RR666, and RR3131).

d For viable CFUs isolated from MIV cultures (none), DNAs were pooled into pairs prior to library construction to reduce sequencing costs.

Donor DNA was either from strain RR3131, a Nov^R^Nal^R^ derivative of the nontypeable clinical isolate 86−028NP (Harrison *et al.* 2005; Mell *et al.* 2011) or the control MAP7 strain (RR666), a multiantibiotic resistant derivative of the recipient Rd strain (Catlin *et al.* 1972). In both cases, the Nov^R^ and Nal^R^ single-nucleotide markers (in the *gyrB* and *gyrA* genes) were >750 kb apart and hence always carried on different molecules in the donor DNA preparations.

Transformed cultures were diluted and plated onto sBHI agar ± antibiotics (2.5 µg/mL for novobiocin and 3 µg/mL for nalidixic acid) to measure transformation frequencies, calculated as Nov^R^ or Nal^R^ antibiotic-resistant colonies per CFU. For MIV cultures, observed Nov^R^Nal^R^ cotransformation frequencies were compared with those expected if all cells were equally competent, (Nov^R^/CFU) * (Nal^R^/CFU), providing estimates of the fraction of competent cells in the culture if competence is a binary on/off cellular state (Goodgal and Herriott 1961; Mell *et al.* 2011).

To permit detection of heteroduplex segregation that had occurred within the original transformed cells, antibiotic-resistant colonies were (a) not restreaked prior to inoculation for sequence analysis and (b) picked from plates with <30 colonies to reduce the chance that individual colonies arose from more than one cell. Picked colonies were inoculated into 5-mL sBHI cultures overnight; 1 mL of each resulting culture was stored in 15% glyercol at −80°, and the remaining 4 mL was centrifuged at 4000*g* for 5 min to collect cell pellets for DNA extracts.

### DNA extractions

Cell pellets were resuspended in 500 µL of resuspension buffer (50 mM Tris-HCl, 50mM EDTA, pH 8), lysed by adding lysis buffer (final concentrations of 1% sodium dodecyl sulfate and 200 µg/mL proteinase K), then incubated at 65° for 60 min. Potassium acetate (pH 8) was added to a final concentration of 1.25M, and the mixtures were centrifuged at 10,000*g* at 4° for 10 min to remove cell debris. Supernatants were precipitated in 1 vol of isopropanol, and pellets were then washed in 70% ethanol, resuspended in 500 mL of TE (10mM Tris-HCl, 1 mM EDTA, pH 8) + 20 µg/mL RNase A, and incubated for 1 hr at 37°. Resuspensions were extracted 2× in 1 vol phenol/chloroform/isoamyl alcohol (24:23:1). DNA was precipitated from the aqueous fractions by addition of 0.1 vol of 3M sodium acetate and 2 vols of 100% ethanol, followed by centrifugation, washing in 70% ethanol for 10 min, and resuspension in 50 µL TE. This typically yielded ~15−20 µg of pure DNA fragments. DNAs were checked for purity and yield by spectroscopy (NanoDrop 1000) and agarose gel electrophoresis, diluted to 100 ng/µL, and arrayed into a 96-well plate for sequencing. DNAs extracted from parental strains RR722, RR3131, and RR666 were included as controls.

A total of 99 genomic DNAs were extracted (Table 1): three control strains, 36 Nov^R^ and 36 Nal^R^ colonies grown from three independent MIV transformations by RR3131 donor DNA, eight Nov^R^ colonies grown from a late-log transformation, and 16 unselected Nov^S^ Nal^S^ colonies grown from MIV transformations. Because these last 16 genomic DNAs were expected to be mostly untransformed, they were pooled 1:1 in pairs prior to library construction, for a total of 91 DNA samples (File S2).

### DNA sequencing and data processing

Methods for sequencing, alignments, and variant calling were a modification of our previous approach (Mell *et al.* 2011), and are detailed in File S1. In brief, sequence reads from each DNA sample were aligned to both donor and recipient genome references, followed by re-alignment of reads with short indels (Table 2) (Li and Durbin 2010; Li *et al.* 2009; McKenna *et al.* 2010; Quinlan and Hall 2010; Thorvaldsdottir *et al.* 2013). Multisample variant calls were generated from the two sets of read alignments, followed by application of stringent filters to define a set of “gold-standard” variants that reliably distinguished donor from recipient—using either reference genome—for both SNVs and SVs (Table 3 and Table 4). Filtering comprised cross-validation between the two control datasets (donor and recipient) and both corresponding reference sequences, as well as exclusion of error-prone positions arising due to systematic sequencing and alignment errors. The filtering also excluded novel alleles absent from either control dataset; such putative new mutations are considered further below. For each sequenced DNA, the genotype at each variant position in the gold standard set was scored as recipient, donor, or mixed (when both alleles were observed). “Donor segments” were defined as contiguous runs of positions with donor-specific variants. Donor segments containing only a single mixed variant (n = 18) were excluded to eliminate probable sequencing artifacts. “Recombination tracts” were defined as clusters of donor segments whose outermost end points fell within 100 kb. A complete listing of all donor segments and their putative clustering is in File S3.

**Table 2:** Sequencing statistics

		Control Reads			Experimental Reads (Remaining 88 Samples)			
Genome*^a^*	Statistic	RR722	RR666	RR3131	Mean	SD	Min	Max
None	QC-passed	2,133,684	2,331,004	2,012,650	1,859,324	705,545	795,476	3,667,922
	% QC-failed*^b^*	45.68	47.81	44.01	48.62	13.96	24.1	72.48
Rd reference	% Aligned*^c^*	99.95	99.94	87.66	99.74	0.24	98.31	99.95
	Median depth*^d^*	55 ± 25	61 ± 27	49 ± 25	49 ± 23	18 ± 12	21 ± 9	94 ± 56
	Unmapped*^e^*	674	1,200	112,011	1,790	1,172	375	5,888
	% Coverage*^f^*	99.81	99.79	91.35	99.68	0.15	99.19	99.89
86-028NP reference	% Aligned*^c^*	92.16	91.76	99.89	91.76	0.22	90.37	92.06
	Median depth*^d^*	56 ± 27	61 ± 27	50 ± 24	49 ± 23	18 ± 12	21 ± 9	94 ± 56
	Unmapped*^e^*	241,928	242,245	1,821	243,056	1,625	236,352	246,081
	% Coverage*^f^*	87.01	87.00	99.65	86.69	0.14	86.44	87.21

QC, quality control; MAD, median absolute deviation

a The sequence reference used for short-read alignment.

b %QC-failed reads accounts for both those that had failed the Illumina chastity filter and those that were removed by the utility sortPairedReads, which identifies and culls read pairs containing the sequencing adaptors. The vast majority of these were adaptor dimers.

c %QC-passed reads mapped indicates how many reads were aligned to the reference genome indicated.

d Median ± MAD read depth across all reference positions supported by at least one read.

e Number of reference positions with no supporting aligned read (read depth = 0).

f Fraction of reference genome positions covered by at least three reads.

**Table 3:** SNVs distinguishing donor from recipient genomes—Filtering and cross-validation

	Rd Reference	86-028NP Reference
Total initial variants detected in 91 short-read datasets*^a^*	40,398	44,591
Short indels*^b^*	818	1228
Ambiguous lift-over position in reciprocal reference*^c^*	1712	5495
Invariant/ambiguous control genotype*^d^*	2251	1074
High-frequency mixed genotype*^e^*	16	162
Invariant/ambiguous genotype at lifted-over position*^f^*	226	1257
Conflict between genotypes in reciprocal alignments*^g^*	312	
Final set of “gold-standard” filtered SNVs*^h^*	35,063	
Transforming SNVs (≥1)*^i^*	10,449	

SNV, single-nucleotide variation.

^*a-f*^Because the sequence reads were aligned to both the donor and recipient genome references, variant calls and filtering were initially carried out independently on the two sets of alignments, one for each reference. This allowed for cross-validation of variant calls and elimination of alignment artifacts.

a Total positions with ≥1 alternate allele out of 91 samples aligned to each parental reference (Rd or 86-028NP). Due to selectable markers introduced in the donor strain, genotypes were manually corrected for MAP7-specific variation prior to counting.

b The number of short indels in ≥1 clones (predominantly simple sequence repeat variants). These were excluded from further analysis as SNVs.

*^c-f^*Progressive filters against error-prone and ambiguous calls; report filtered positions that passed the previous filter.

c Positions where whole-genome alignment gave two different lift-overs (conversions between recipient and donor coordinates), depending on which reference was used as the query during Mauve alignment.

d Positions where parental base calls were invariant or ambiguous (excluding*^c^*). The expected pattern is that donor reads would have the reference base against 86-028NP and an alternate base against Rd, while the recipient reads would have the reciprocal.

e Positions where >5% of samples gave an ambiguous/mixed call (excluding*^c-d^*), removing most error-prone positions but not mixed genotypes arising from transformation.

f Positions in which the lift-over position (coordinate the reciprocal alignment) were invariant or ambiguous (excluding *^c-e^*), reconciling the variant positions between the two alignments.

g Positions passing the above filters,*^c-f^* but with ≥1 conflict in the base call made depending on the reference used.

h Final set of filtered SNVs. All positions have a valid lift-over to the reciprocal reference, a low frequency of ambiguous/mixed genotypes, and consistent genotypes between both parental control reads and reciprocal alignments of the parental references.

i Count of SNVs for which ≥1 of the 72 selected recombinants had a donor allele.

**Table 4:** SVs distinguishing donor from recipient—Filtering and cross-validation

Class	All SVs*^a^*/Filtered SVs*^b^*			Transforming SVs (≥1)*^c^*		
	Deletes*^d^*	Inserts*^e^*	Total*^f^*	Deletes*^d^*	Inserts*^e^*	Total*^f^*
1 bp	277 / 166	318 / 203	595 / 369	43	46	89
2−10 bp	192 / 154	180 / 148	369 / 300	33	29	61
11−100 bp	53 / 34	63 / 51	95 / 68	5	12	14
101−1000 bp	39 / 33	34 / 30	90 / 73	4	5	12
>1000 bp	23 / 21	30 / 18	62 / 48	5	4	10
Complex*^g^*			10			0
Total	584 / 408	625 / 450	1221 / 868	90	96	186

SVs, structural variants; IGV, Integrative Genomics Viewer.

a Total SVs from a Mauve alignment. Indel directionality is relative to transformation, such that insertions are donor-specific and deletions are recipient-specific. Reporting indels is complicated by “insertional deletions,” where donor sequences would replace recipient sequences (17% of filtered SVs), a pattern more common for large SVs (44% affecting >100 bp), so net change is reported.

b Subset of indels for which reads distinguished donor from recipient and <5% of genotypes were ambiguous.

c Subset of SVs with the donor allele present in ≥1 of the 72 selected clones.

d SVs with indicated number of bps would be deleted by transformation; the net deletion (*i.e.*, the donor allele was shorter than the recipient allele).

e SVs with indicated number of bps would be inserted by transformation; the net insertion (*i.e.*, the donor allele was longer than the recipient allele).

f SVs affecting the indicated number of bps; sum of donor and recipient allele lengths.

g Complex SVs (inversions, relocations, *etc*.) missed by the genotyping method but manually inspected using IGV.

### Data analysis and statistics

Plotting, curve fitting, and linear modeling used the R statistical programming language, including the fitdistrplus package. The Kolmogorov-Smirnov statistic (*D*) was used to evaluate goodness-of-fit (K-S test). Permutation tests were used to generate random distributions of donor segment locations in order to calculate the probability of overlapping donor segments between different clones. Differences between the size of breakpoint intervals and genome-wide SNP spacing were evaluated by K-S test. Fisher’s exact tests were used to compare transforming insertions to transforming deletions and to compare breakpoint classes with their expected distributions. Student’s *t*-tests (two-tailed, paired) were used to evaluate differences in transformability between strains.

### Polymerase chain reaction (PCR) validation of donor segments in DNAs with mixed genotypes

For one of the samples with two mixed segments (RR4074), allele-specific PCR was used to confirm that the original sequenced DNA sample contained distinct cell populations with either the donor or recipient versions of both segments. The stored strain was streaked and individual colonies containing each of the two genotypes were identified by colony PCR. Because RR4107 and RR4108 were sequenced as a pool, PCR was also used to confirm which clone contained the observed recombination tract. Primers used are in Table S1.

### Mapping a transformation frequency modifier

Each of the 96 sequenced strains, along with donor and recipient controls, was initially assayed for transformability using a “transformation-during-growth assay,” in which exponentially growing cultures at OD_600_ = 0.2 were diluted 1/100 in sBHI containing 10 µg/mL MAP7 DNA, followed by incubation at 37° for 8 hr, dilution, and plating onto sBHI agar ± kanamycin (7 µg/mL). Candidate recombinants, including RR4108, were reassayed with the MIV starvation method. Radiolabeled DNA uptake assays were performed as described (Maughan and Redfield 2009a).

Deletions of the *comM* gene (HI1117) were generated in three distinct strain backgrounds (RR722, RR3131, and RR4108). Construction of the RR3131 *comM*::spec and RR4108 *comM*::spec strains used transformation by a 4.25-kb amplicon across the *comM*::spec allele generated from a previously characterized *comM*::spec deletion made in the RR722 background (RR3121)(Sinha *et al.* 2012) (primers *comM*-F and *comM*-R in Table S1). Mutants were selected on 200 µg/mL spectinomycin and confirmed by PCR and Sanger sequencing.

The Rd and 86-028NP alleles of *comM* also were cloned into the *Eco*RI site of the low-copy vector pSU20 (Bartolome *et al.* 1991; Poje and Redfield 2003a) with the native promoter to generate pRd*comM* and pNP*comM*; the gene was amplified with ~700-bp flanks from the appropriate genomic DNA using primers *comM*_*Eco*RI_F and *comM*_*Eco*RI_R (Table S1), and electroporated into the RR722 *comM*::spec and RR4108 *comM*::spec strains. Complementation assays of the resulting strains were carried out with wild-type controls, measuring the Kan^R^ transformation frequencies from MIV-induced cultures. The gene map was made with the assistance of GenoPlotR (Guy *et al.* 2010).

### Data deposition

Sequence data for each sample were deposited—to the NCBI short-read archive under project accession SRP036875—as BAM files reporting alignments to the Rd (KW20) genome reference, with filename prefixes according to File S2. Deposited read alignments (Illumina 8.1+ quality encoding) were purged of paired reads failing Illumina chastity filters or containing adaptor sequences, but unaligned reads were retained to allow realignment to the 86-028NP donor genome reference.

## Results

### Summary of recombinant clone isolation and genome sequencing

*Haemophilus influenzae* recipient cultures of the lab strain Rd were transformed with donor genomic DNA from a derivative of the clinical isolate 86-028NP carrying two antibiotic resistance markers (Nov^R^ and Nal^R^). The divergence between the strains provides a high density of genetic markers; the donor and recipient genomes differ at 2.34% of alignable nucleotide positions (at SNVs), and ~10% of each genome is absent from the other (at indels and other rearrangements, *i.e.*, SVs), typical for a pair of *H. influenzae* isolates (Harrison *et al.* 2005; Hogg *et al.* 2007; Mell *et al.* 2011).

Colonies were propagated from four transformation experiments (Table 1), and their purified DNA was short-read sequenced on an Illumina HiSeq to give nearly complete identification of every nucleotide in each genomic DNA sample (Table 2 and Figure S1). Median read depth per position was ~50-fold, and although variation in read depth along the genome was high, this variation was consistent between libraries (Figure S2). Short-read sequence datasets were processed using an improved and expanded version of our previous approach (Mell *et al.* 2011), which now incorporates genotype calling at SVs, as detailed in the section *Materials and Methods*. This provided a comprehensive genotype table for all the sequenced chromosomes at 35,063 SNVs and 868 SVs, 121 of which affected ≥100 bp (Table 3 and Table 4).

### Global pattern of transformation in selected recombinants

Three of the four transformation experiments used the standard competence-induction protocol, in which exponentially growing cells are transferred to the starvation medium MIV to induce maximal competence, followed by incubation with donor genomic DNA (Table 1). From each transformation, 12 Nov^R^ and 12 Nal^R^ recombinant colonies were propagated for sequencing (72 in all). The antibiotic resistance selection ensured that each sequenced clone had acquired at least one donor segment, precluding inadvertent resequencing of untransformed recipient chromosomes.

**Figure 2:**
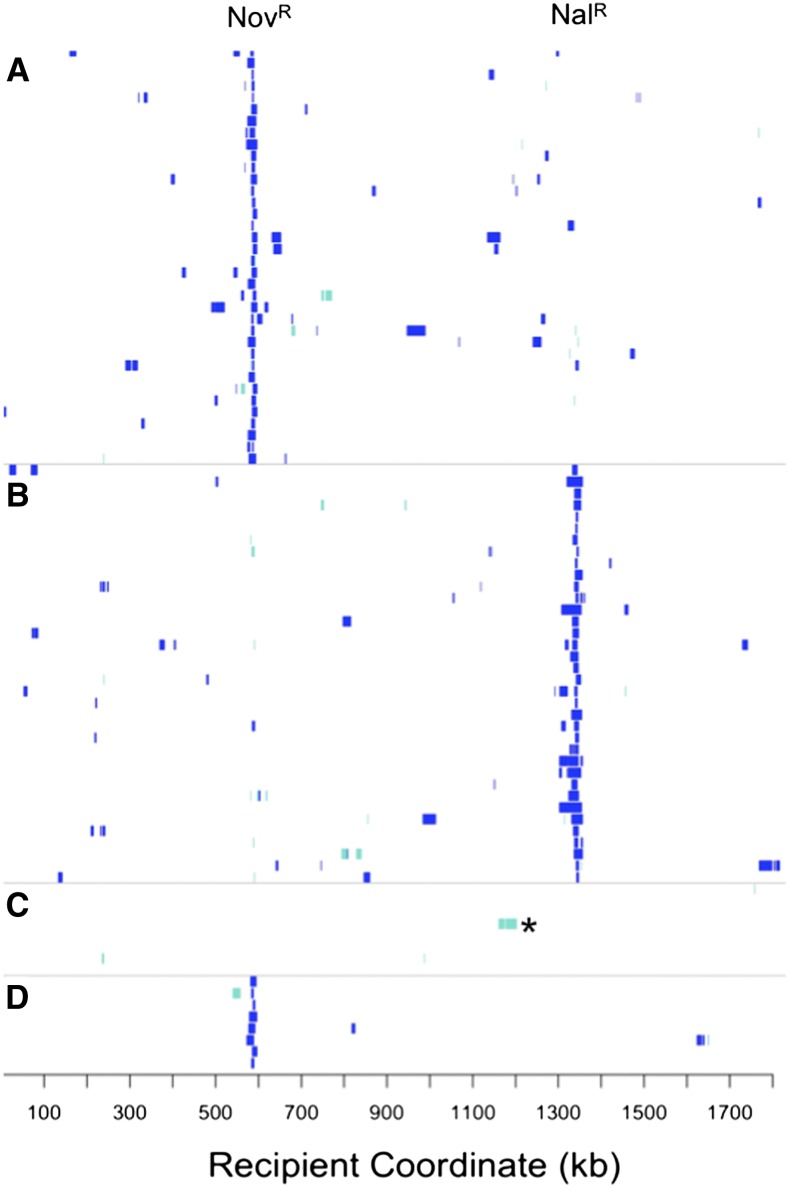
Extent of recombination in 96 transformed colonies. Each row indicates a genome, with coordinates are according to the recipient Rd genome sequence. Blue hatches indicate donor-specific SNVs. Turquoise hatches indicate a mixture of donor- and recipient-specific SNVs. (A) 36 Nov^R^-selected recombinants from MIV transformations. (B) 36 Nal^R^-selected recombinants from MIV transformations. (C) Eight pools of two unselected clones from MIV transformations (such that turquoise hatches indicate donor variation in one of the two pooled clones). *Indicates the recombination tract in RR4108 that confers altered transformability (see text). (D) Eight Nov^R^-selected recombinants from a late-log transformation. SNV, single-nucleotide variant.

**Figure 3:**
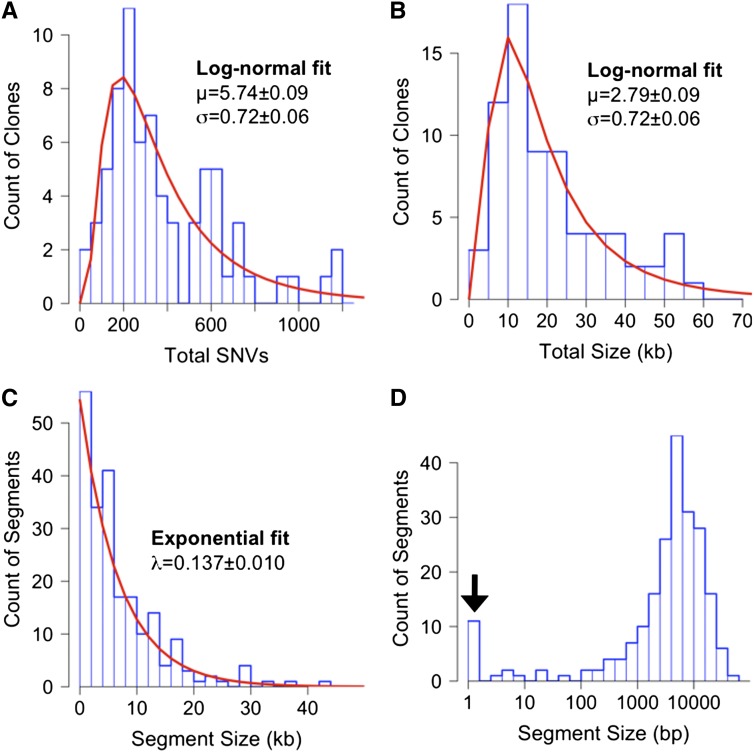
Summary histograms of transformation in the 72 selected colonies. (A) Total donor SNVs; (B) combined length of donor segments in each clone (measured from outermost donor-specific SNVs aligned to the recipient genome); (C) length of individual donor segments; (D) same as in (C), but with segment length on a log-scale and the 11 donor segments containing only one SNV are indicated with an arrow. Best fits are shown as red lines in (A−C) used distributions that best fit the data, as determined using the R package fitdistrplus [log-normal for (A−B) and exponential for (C)]. “Mixed” segments were included in these measurements. SNV, single-nucleotide variant.

Modeling the process of natural transformation for population genetic studies requires that the statistical distribution of recombination events be determined. The 72 selected recombinants carried extensive donor genetic variation in their chromosomes in 216 long contiguous segments, with each clone carrying at least one donor segment spanning the expected antibiotic resistance allele (Figure 2, A and B and File S3). The total extent of recombination per clone was consistent with a lognormal distribution (Figure 3, A and B; K-S test for total donor SNVs: *D* = 0.073, *p*-value = 0.84; and for total kb replaced: *D = 0.049*, *p*-value = 0.99). Individual recombinant chromosomes contained on average 394 donor SNVs in 1−10 donor segments, collectively replacing between 2.7 and 58.5 kb of the recipient chromosome, or on average 1.13% of genome length. This overall extent of recombination was approximately twofold higher (mean 1.13% genome length) than that predicted from the transformation frequencies of the Nov^R^ and Nal^R^ markers alone (mean 0.58% per CFU, Table 1), suggesting that these particular markers might transform at lower rates than the genome average.

### Donor segment properties

The average length of the 216 donor segments was 6.9 kb (median 4.8 kb), when defined by their outermost donor-specific variants (breakpoint intervals themselves are analyzed below). Segment lengths followed an exponential distribution (Figure 3C; K-S test for all donor segments: *D* = 0.076, *p*-value = 0.16), with the largest segment 43.1 kb long, or 2.4% of the genome length,. The exponential fit to the distribution of segment lengths was improved by excluding the eleven outliers that contained only a single SNV (indicated by the arrow in Figure 3D; K-S test for donor segments containing >1 SNV: *D* = 0.053, *p*-value = 0.62), suggesting that these arise by a distinct process. These and other very short donor segments are discussed below as potential remnants of donor-directed mismatch repair. These results with *H. influenzae* contrast with the shorter recombinant segments detected in *Streptococcus pneumoniae* and *Helicobacter pylori* transformants (Croucher *et al.* 2012; Lin *et al.* 2009). This suggests that mechanistic differences in uptake (or subsequent steps) might favor recombination of longer segments in *H. influenzae*, although importantly, variation in average segment length within species has not been evaluated.

**Figure 4:**
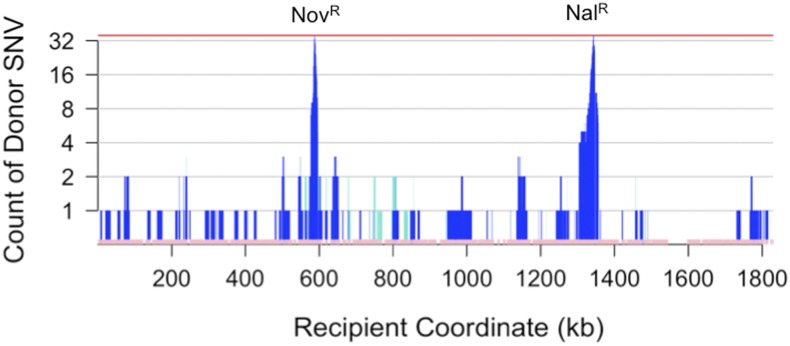
Cumulative SNV transformation. Count of donor-specific (dark blue) and mixed (turquoise) SNVs across the 72 selected transformants illustrated in Figure 2, A and B (y-axis) using the recipient genome coordinate (x-axis). Bottom pink track shows all 35,063 SNV markers used to distinguish donor from recipient. Regions with low marker density correspond to recipient-specific insertions, *e.g.*, a recipient-specific prophage insertion at ~1600 kb. SNV, single-nucleotide variant.

In 8 colonies, 13 “mixed” donor segments were observed where the base at each SNV was supported by sequence reads of both donor and recipient alleles (to exclude sequencing artifacts, “mixed” segments had to consist of >2 SNVs) (turquoise segments in Figure 2, A and B, and Figure 4A). These mixed segments were contiguous runs of 9−157 variant positions with mixed donor/recipient base calls (0.9−13.7 kb), which could not have arisen by sequencing error or cross-contamination of DNA samples. Instead they most likely arose by heteroduplex segregation, as discussed below.

### Total recombination across the selected recombinants

Transformation at unselected sites was sufficiently high that—considering all 72 recombinants—29.8% of all “gold-standard” donor SNVs were present in at least one recombinant (Table 3 and Figure 4), predicting that a set of several hundred independent recombinants could potentially contain most of the donor-specific variation in short discrete intervals.

No obvious transformation hotspots were observed; two overlapping segments were seen at only six intervals distant from the selected sites (see below), and three overlaps at only one interval (at ~1150 kb in Figure 4). As a control, random shuffling of donor segment locations found 3 overlapping segments in <5% of 1000 permutations. However, a long interval devoid of donor segments was observed at ~1500−1700 kb, possibly indicating a transformation coldspot (Figure 4). Notably, this interval is roughly opposite the predicted origin of replication at ~603 kb (Salzberg *et al.* 1998). These results do not rule out variation along the genome in transformation frequency (as observed in Ray *et al.* 2009), since the power to detect hotspots and coldspots in this study was limited by the number of clones sequenced.

### Local pattern of recombination around the selected sites

**Figure 5:**
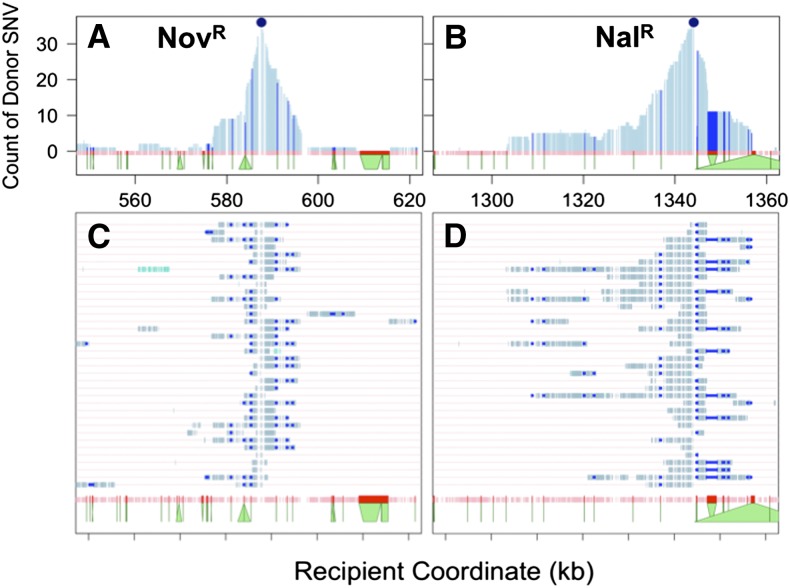
Cotransformation around selected sites. (A) and (B) show zooms around the Nov^R^ and Nal^R^ selected sites for Nov^R^ and Nal^R^ clones, respectively, whereas (C) and (D) draw the genotype for each specific clone. Donor SNVs are shown in light blue, whereas donor SVs are shown in dark blue (following the width of the recipient allele). The bottoms of the green trapezoids along the x-axis show the width of the SVs according to the donor genome. For example, the SV at ~584 kb is a 2.7-kb transposon specific to the donor strain, and the SV at ~1347 kb has 2 kb in the recipient that do not align with 300 bp in the donor. SNV, single-nucleotide variant, SV, structural variant.

Selection for a single donor SNV (Nov^R^ or Nal^R^) resulted in clones with extensive cotransformation of adjacent donor-specific variation (Figure 2, Figure 4, and Figure 5). If transformation frequency were uniform across the genome, the donor allele frequency would be expected to decrease exponentially with increasing distance from the selected site (Croucher *et al.* 2012), but this was not seen. Instead we observed two distinct phenomena flanking the selected sites: (a) intervals of reduced cotransformation at or near positions of structural variation (indels and other rearrangements), and (b) a markedly skewed distribution of cotransforming donor alleles to one side of the Nal^R^-selected site within the *gyrA* locus.

Local reductions in transformation around the selected sites often corresponded to positions of indels (trapezoids in Figure 5, A and B), suggesting that the unrecombined heterologous DNA embedded within (or at the edge of) longer heteroduplex molecules is trimmed out by nucleases after strand exchange (Mell *et al.* 2011) (discussed in the next section). In Figure 5B, the strong skew of donor allele frequency to one side of the Nal^R^ selected clones suggests that transformation is promoted by some sequence factor to the left of the Nal^R^ marker, while transformation at the other end is limited by the presence of a 23-kb prophage specific to the donor strain (right-hand triangle).

### Clustering of donor segments into “recombination tracts”

As observed in previous work in several organisms, donor segments in individual recombinants were often clustered near other segments, suggesting that endonucleases disrupt long DNAs after uptake (Figure 2 and Figure 5, C and D) (Croucher *et al.* 2012; Lin *et al.* 2009; Mell *et al.* 2011). To estimate the number of independent transformation events in each genome, we treated clustered segments as “recombination tracts” derived from the same donor molecule, using as an upper limit a total span of <100 kb (consistent with the length of DNA in high molecular weight preps and the high frequency of cotransformation around the Nov^R^ and Nal^R^ alleles). Although this 100 kb cut-off is somewhat arbitrary, it is likely generous, since defining recombination tracts in this way gives a mean tract size of 19.5 kb (median 12.9 kb), and only 16 of the 130 tracts (collapsed from 216 segments) exceeded the size of the largest single uninterrupted donor segment (43 kb; segment #71 in File S3). However, because the original taken up DNA fragments are unknown, it is important to note that some apparent clustering may instead result from coincidental transformation by two independent fragments. Clustering was determined using donor rather than recipient genome coordinates, since rearrangements between the two genomes otherwise caused the spurious appearance of clustering or independence of segments in several clones (Figure S3 and Figure S4).

**Figure 6:**
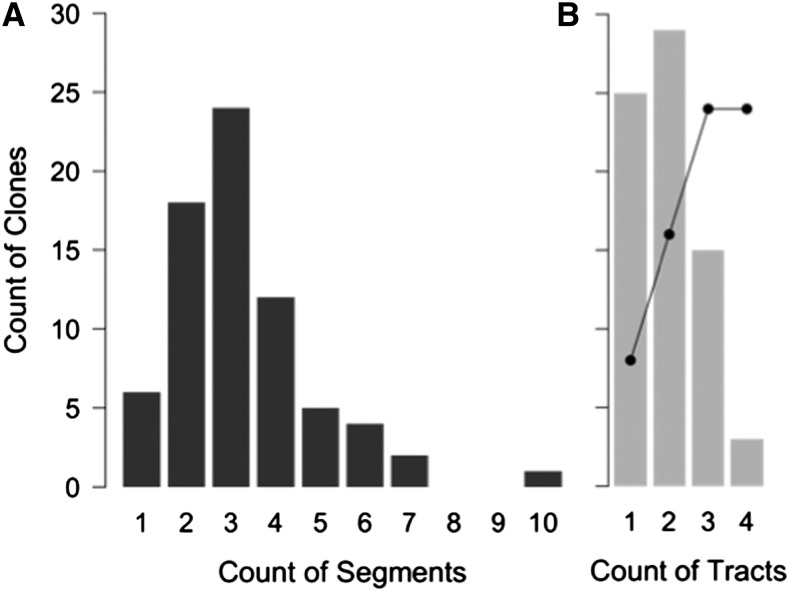
Estimates of maximal donor segment clustering. Histograms are shown of (A) donor segments detected per clone, (B) minimum “recombination tracts” per clone, defined as sets of adjacent segments falling within 100-kb windows. The black line estimates the number of independent recombination events in the original competent cells, assuming heteroduplex segregation and a maximum of four events.

Consolidation of segments into tracts allowed us to estimate the minimum number of independent transformation events in each recombinant and to distinguish properties of selected tracts from those that were acquired independently of the selected loci. Twenty-five of 72 colonies (35%) had a single recombination tract spanning the selected Nov^R^ or Nal^R^ locus, whereas the remainder had 1-3 additional tracts (Figure 6). Notably, if segregation of heteroduplex recombination products is the norm, then the extent of transformation in these genomes underestimates by twofold the extent of donor strands recombined at unselected sites in the genomes of individual competent cells. These would have a ~50% chance of segregating to an antibiotic-sensitive cell and thus be missed by this study, depending on which donor strands were originally translocated and recombined (Figure 1).

Based on the observed number of tracts per recombinant, the line in Figure 6B estimates how many independent molecules the original competent cells had taken up and recombined into their chromosomes (recombination events), given the assumptions that: (a) the probability of each event is independent of the others, (b) each event produces heteroduplex that segregates to two cells, and (c) cells have a maximum of four events. This last assumption simplifies the calculation; since it is conservative the actual distribution may instead be shifted further to the right. This analysis suggests that ≥89% of cells selected for transformation at a single locus had sustained at least one additional recombination event.

### No apparent effect of nucleotide divergence on limiting donor segment location

As we previously observed, the Nov^R^ and Nal^R^ alleles transformed strain Rd about threefold more efficiently when the donor background was Rd than when the donor background was the diverged 86-028NP (Mell *et al.* 2011) (Table 1, *p*-value = 0.024, 1-tailed paired *t*-test). The lower transformation frequency with DNA from a diverged background is presumably due to sequence heterology around the selected alleles, which could either reduce the probability of strand exchange or increase the probability of recipient-directed heteroduplex correction.

**Figure 7:**
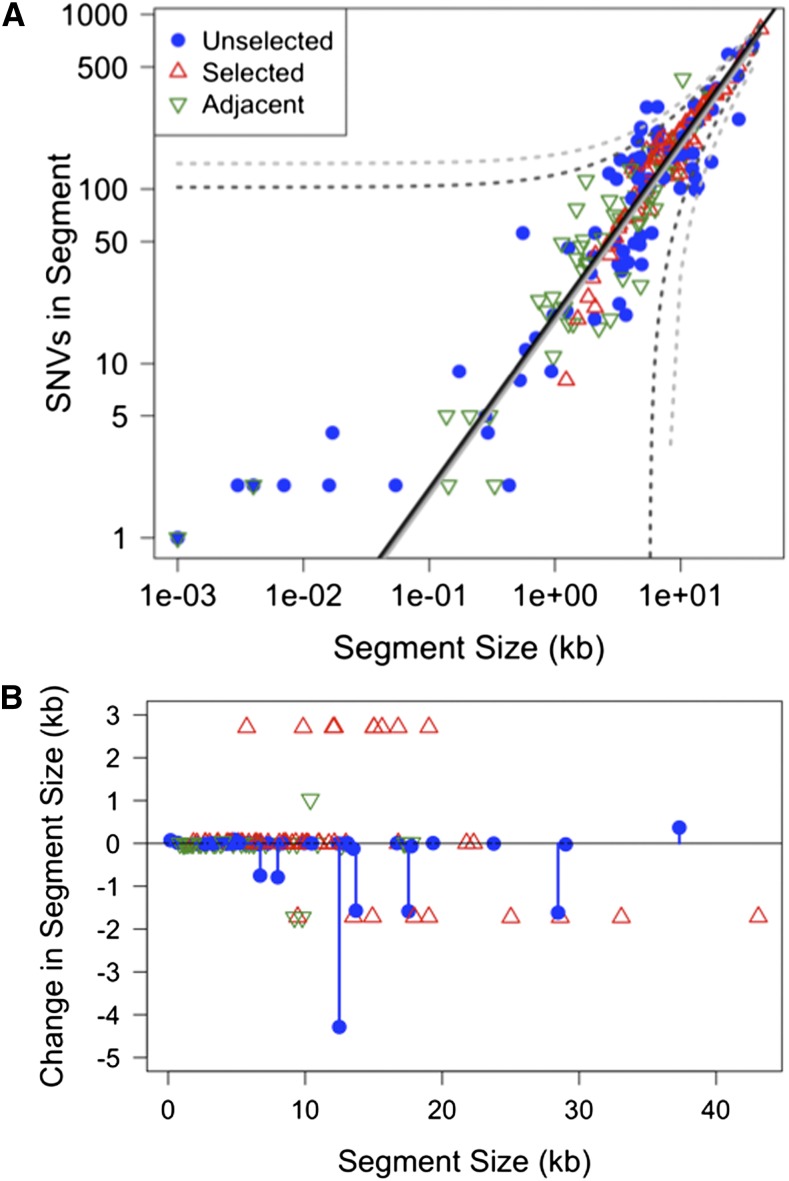
Effects of sequence divergence. (A) Nucleotide divergence has no observable effect. Plot shows the count of donor SNVs in each segment as a function of segment size. Colored symbols indicates whether the segment spanned either the Nov^R^ or Nal^R^ selected site (red triangles), was in the same tracts as a selected segment (green triangles), or was considered independent of the selected segment (blue circles). Black line indicates genome-wide average. Solid and dotted lines indicate the linear fit and 95% prediction intervals for all segments (dark gray) and only segments more than 100 kb from the selected site (light gray). (B) Indels/SVs changed length of the recombined segments. The x-axis shows length of recipient sequences replaced by each donor segment, while the y-axis shows the change in length between the recipient and donor segments. Color-coding as in (A), with blue lines to highlight unselected segments. The recurrent 2.4-kb insertion and 1.7-kb deletion correspond to the SVs adjacent to the Nov^R^ and Nal^R^ sites, as described in the example in Figure 5.

Although this result predicts that donor segments would be preferentially found in parts of the genome with lower sequence divergence, their locations showed no obvious correlation with local nucleotide identity between the donor and recipient genomes (Figure 7A). Instead, the number of donor SNVs per segment was linearly correlated with donor segment length (R^2^ = 0.92), closely matching the genome-wide mean SNV density (superimposed solid lines in Figure 7A; 19.3 SNVs/kb genome average *vs.* a linear estimate of 18.1 ± 0.3 SNVs/kb in donor segments). The lack of effect of heterology was not an artifact of selection for the Nov^R^ and Nal^R^ alleles, since it held even when only donor segments >100 kb from the selected sites were considered (Figure 7A; linear estimate of 17.3 ± 0.8 SNVs/kb; R^2^ = 0.85). This result is consistent with recent observations in *Streptococcus pneumoniae*, as well as with the finding in *H. influenzae* that individual donor segments span regions of both high and low nucleotide divergence (Croucher *et al.* 2012; Mell *et al.* 2011).

### Transforming indels

Although SVs (insertions, deletions, and other rearrangements) often acted as blocks to recombination progression, they also sometimes transformed as parts of longer recombination tracts (Figure 5). Of the 868 gold-standard SVs, 186 were found in ≥1 recombinant (21.4% of the total), including 22 that affected >100 bp (Table 4). All transforming SVs were accompanied by co-transforming SNVs, and were thus parts of longer recombination tracts. Complex SVs—which included 10 inversions and relocations—did not transform any of the 72 recombinants.

One effect of transforming SVs is to change the length of the replaced recipient segment (Figure 7B). Many length changes were caused by indels flanking the selected Nov^R^ and Nal^R^ variants (Figure 5), so transforming SVs within selection-independent donor segments were examined separately to test whether there were biases toward deletion or insertions. For unselected segments, seven segments had a net deletion of >100 bp, but only 1 had a net insertion of >100 bp (Figure 5, blue lollipops), although the donor and recipient genomes are distinguished by roughly equal numbers of insertions and deletions (Table 4). This difference was not significant by Fisher’s exact test (*p*-value = 0.14), but a similar pattern was reported for *S. pneumoniae* transformants (also not significant) (Croucher *et al.* 2012). Such a bias need not be intrinsic to the mechanism of recombination, but could occur because, for a given length of recombining DNA, donor-specific insertions have less flanking homology than donor-specific deletions. This predicts that in the absence of selective forces, ‘accessory loci’ of the pan-genome might be more likely be lost than gained by transformation, depending on the size distribution of donor DNA (Croucher *et al.* 2012).

### Donor segment breakpoint intervals are slightly longer than expected but flank more indels

**Figure 8:**
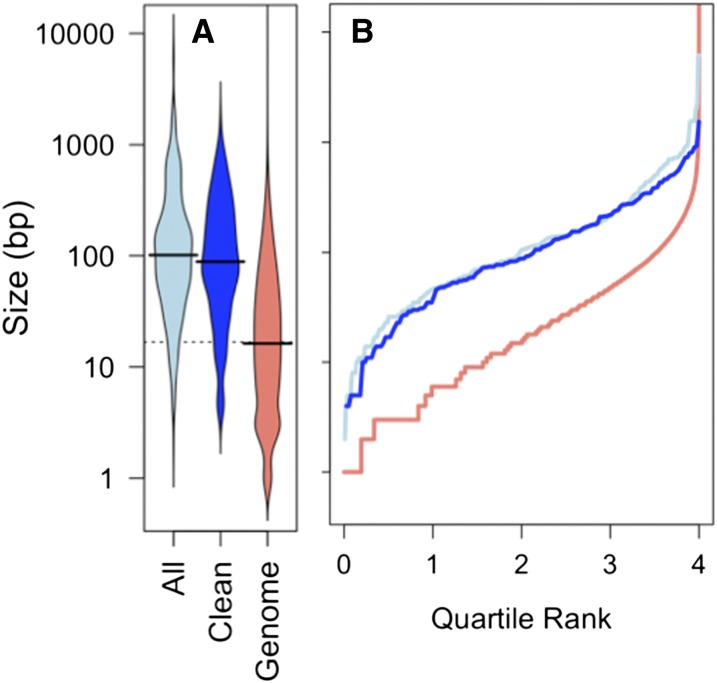
Breakpoint interval lengths are longer than genome average. (A) Beanplot of sizes of breakpoint intervals (distance between outermost donor-specific SNVs and innermost recipient-specific SNVs): “All” includes all breakpoint intervals; “Clean” includes only unique breakpoints of unselected donor segments unaffected by SVs; and “Genome” includes all intervariant spacings (35,931 total). Black lines show the medians; width of bean is proportional to the density of values with that spacing. (B) Plot of the same data, where the x-axis is the cumulative proportion of the data with interval size less than the value indicated on the y-axis.

We defined donor segment intervals by the outermost variants of contiguous runs of donor-specific alleles, but the true recombination breakpoints must lie between these positions and their nearest flanking recipient-specific alleles. Because the lengths of breakpoint intervals are also measures of local sequence identity, we tested whether they were distinct from the genome-average variant spacing. Figure 8 shows that breakpoint intervals were significantly longer than the average spacing between variants (median 106 bp, compared with 15 bp for genome-wide variant spacing; K-S test: *D =* 0.50, *p*-value << 0.001). This difference was seen, even for subsets of the data that included only breakpoint intervals for unselected donor segments (>100 kb away) and when only unique breakpoint intervals were used (median 92.5 bp and 87 bp, respectively).

The high sequence identity at breakpoints likely underestimates the effect of sequence divergence on recombination breakpoints, since many were likely to have been pre-determined by the donor fragment ends or by degradation of ssDNAs after uptake. The connection between high local identity at recombination breakpoints—yet no connection with limiting the locations of donor segments themselves—implies that initiation of strand exchange from the ends of RecA-coated donor DNA may require higher levels of sequence identity than the subsequent progression of strand exchange, consistent with biochemical analyses of RecA-mediated strand exchange (Gupta *et al.* 1998; Kowalczykowski and Eggleston 1994).

In contrast, breakpoint intervals were ∼fourfold more likely to have a SV as the nearest recipient-specific variant (Table 5): 10.9% of breakpoints had a flanking recipient SV (47 of 432 total), whereas only 2.4% of genome-wide intervals are flanked by SVs (Fisher’s exact test *p*-value << 0.001). Only part of this effect is due to recurrent breakpoints at the same SV (Figure 5), since enrichment of SVs at breakpoints was still seen even when only unique breakpoint locations and/or only unselected segments were considered (*p*-values << 0.001). This enrichment could arise because strand exchange frequently terminates at SVs, or because heteroduplex molecules with mismatched SVs are more efficient targets for heteroduplex correction.

**Table 5:** Classification of recombination breakpoint intervals

	No Flanking SV	Flanking SV	% SV
Genome-wide intervals	35,063	868	2.4
Breakpoint locations	346	27	7.2
Total breakpoints	385	47	10.9
Unselected locations	154	15	9.7
Unselected breakpoints	157	15	9.6

SVs, single variants.

### Evidence of heteroduplex segregation

We observed 13 mixed donor segments in eight recombinants, with SNVs within each segment consisting of ~25–75% reads supporting the donor allele and the remainder supporting the recipient allele (turquoise bars in Figure 2 and Figure 4). The experimental design facilitated recovery of colonies consisting of two genotypes that arose by heteroduplex segregation at unselected sites (Figure 1C); cell were plated shortly after transformation and at low density to limit the risk of independent transformants forming mixed colonies. Consistent with their origin as segregants from a single transformant, each of the colonies with a mixed-genotype segment also had a normal unmixed segment containing the selected Nov^R^ or Nal^R^ allele (Figure 2 and Figure 4); if two transformants had contributed to the same colony, their independent recombination breakpoints would be unlikely to be the same at both ends (see Figure 5), which would leave mixed base calls flanking the two independently selected segments. The origin of one of the selected colonies from two distinct genotypes was confirmed by allele-specific PCR from individual colonies streaked from the stored culture. Strikingly, all but one of the mixed segments are within 200 kb of the predicted origin of replication at ~603 kb (Salzberg *et al.* 1998), suggesting that most mixed tracts arose by segregation of recombination products at unselected loci after DNA replication but before DNA replication through the selected loci, as diagrammed in Figure 1C.

### Translocation and recombination of individual fragments

**Figure 9:**
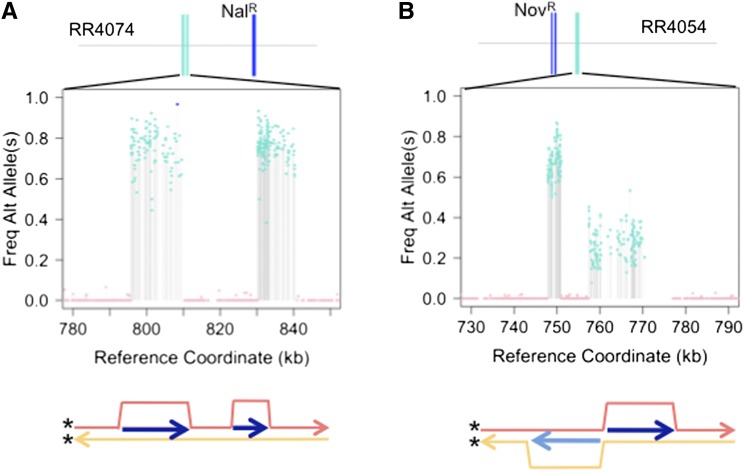
Transformants with evidence of clustered heteroduplex donor segments. Top panels show illustrations of each recombinant. Middle panels shows zoom on the clustered “mixed” donor segments, with the y-axis showing the donor-specific allele frequency. Bottom panel illustrates the inferred recombination intermediate. (A) Heteroduplex segregation of two molecules that transformed the same recipient strand. Clustering suggests a single long strand was translocated into the cytoplasm, but endonuclease activities disrupted the ssDNA before or during recombination. (B) Heteroduplex segregation of two molecules that transformed complementary recipient strands. If derived from a single molecule, each end of the periplasmic dsDNA would have been independently translocated. Since translocation occurs by transport of ssDNA by its 3′-end, translocation from both ends through two different pores would yield two cytoplasmic ssDNAs adjacent to each other, but complementary to opposite strands. Figure S4 shows a clone without mixed tracts that would also have required double-translocation across an inversion, if the clustered segments were derived from the same molecule. ssDNA, single-stranded DNA.

More details were revealed by analysis of allele frequencies in the two colonies that contained clustered segments with a mixed genotype. In one of these colonies (Figure 9A), the two clustered segments both had their donor alleles present in ~80% of reads, consistent with both occurring in the same one of the two subclones, and thus with both originating from the same translocated DNA strand (recombination *in cis*). This was confirmed by allele-specific PCR from colonies streaked from the stored culture. In the other mixed colony (Figure 9B), the two clustered segments had complementary donor allele frequencies (~30% or ~70% of reads). Although this recombination *in trans* could be coincidental translocation of independent fragments complementary to opposite strands, the close spacing of the two segments suggests the more intriguing possibility that both strands of a single long dsDNA fragment in the periplasm were translocated, one from each end. The unmixed donor segments of an additional colony (Figure S4) are also consistent with double translocation of a single donor DNA, in this case spanning an inversion breakpoint.

### Evidence of heteroduplex correction

For 11 donor segments only a single unambiguous donor-specific SNV is present; 7 of these are not clustered into longer recombination tracts. These likely represent genuine outcomes of transformation, rather than being artifacts (either new mutations or sequencing errors that coincidentally produced a donor-specific SNV). Two analyses suggest that the frequency such artifacts is expected to be very low. First, no new high quality variants (alleles not present in either parent) were found in the 72 recombinants, suggesting that new mutations are uncommon. Second, only 10 high-quality variants were identified by sequencing another lab strain, MAP7, an Rd derivative resistant to seven antibiotics (Catlin *et al.* 1972), and only 3 of these are not accounted for by its antibiotic resistances (Table 6).

**Table 6:** MAP7 SNVs

Position	Resist	Gene	Mutation	Codon	Site	Substitution
537,227	Rif^R^	HI0515 (*rpoB*)	C→T	619	1	Ala→Thr
537,378		“	G→T	568	3	Asn→Lys
587,579*^a^*	Nov^R^	HI0567 (*gyrB*)	G→T	140	1	Gln→Lys
587,846*^a^*		“	C→A	51	1	Ala→Ser
587,969*^a^*		“	C→A	10	1	Gly→Cys
597,885	Kan^R^	HI0579 (*fusA*)	G→C	655	2	Pro→Arg
600,806	Str^R^	HI0581 (*rpsL*)	T→C	43	2	Lys→Arg
839,913	Spc^R^	HI0778 (*rplD*)	G→C	65	2	Gly→Ala
935,046		HI0883 (unk)	A→C	356	3	*Gly*→*Gly*
1,344,100*^a^*	Nal^R^	HI1264 (*gyrA*)	C→A	88	1	Asp→Tyr

SNVs detected in MAP7 reads against the Rd reference that were also SNVs in the recipient reads, as well as positions with ambiguous/mixed genotype calls, are not reported. SNV, single-nucleotide variation.

a These variants are also present in the donor RR3131 (86-028NP Nov^R^ Nal^R^)

Because these singlet segments are clearly more abundant than predicted by the overall distribution of donor segments (Figure 3D), they likely arise by a distinct process. We posit that singlet donor segments are best explained by donor-directed mismatch repair from a recombination tract that was subsequently lost by heteroduplex segregation.

### Only a subset of cells in competent cultures is transformed

Observed cotransformation frequencies of Nov^R^ and Nal^R^ were ~10-fold higher than predicted by the product of their single marker transformation frequencies (6.2- to 14.5-fold, Table 1), and these values were consistent for two donor DNAs, both the RR3131 donor DNA and DNA from an Rd-derived strain MAP7 (RR666) (*t*-test *p*-value = 0.26). This excess of double transformants (termed congression) is usually assumed to reflect the presence of noncompetent cells in the culture, and suggests that only ~10% of cells actively took up and recombined donor DNA into their chromosomes (Goodgal and Herriott 1961).

To directly test whether congression measures % competence, we sequenced 16 unselected clones from the MIV transformations. Only 2 of 16 had been transformed, each carrying a single recombination tract, consistent with congression predictions of ~10% competent cells (Figure 2C, File S3). The sizes of the two tracts fell within the range seen in selected clones (2.7 kb and 40.2 kb). The estimates in Figure 6B also reject the null hypothesis that all cells are equally competent, since they predict that 65% of unselected colonies would have recombination tracts (Fisher’s exact test *p*-value = 0.01).

Cultures grown to high density in rich medium (“late-log”) are ~100-fold less transformable than MIV-induced cultures (Redfield *et al.* 2005). To clarify whether competent cells in such cultures are rare or just weakly transformable, a late-log culture was transformed with donor DNA as described previously, giving Nov^R^/CFU of 1.15 × 10^−5^ (Table 1), and 8 Nov^R^ transformants were sequenced. The donor segments of these clones were comparable in size, number, and distribution to those of the MIV-selected transformants and included one ‘mixed’ segment (Figure 2D, File S3, segments averaged 8.6 ± 5.8), suggesting that transformation in late-log cultures results from a very small fraction of fully competent cells. This conclusion is also supported by co-transformation data from late-log cultures, which indicates ~0.1% competent cells (data not shown).

Although we could not rule out subtle differences or stochastic variation in level of transformability these data are consistent with competence induction being an on/off response, in which the starvation conditions of MIV induce a much higher proportion of cells to become competent than late-log conditions.

### Natural variation in transformability is polygenic

**Figure 10:**
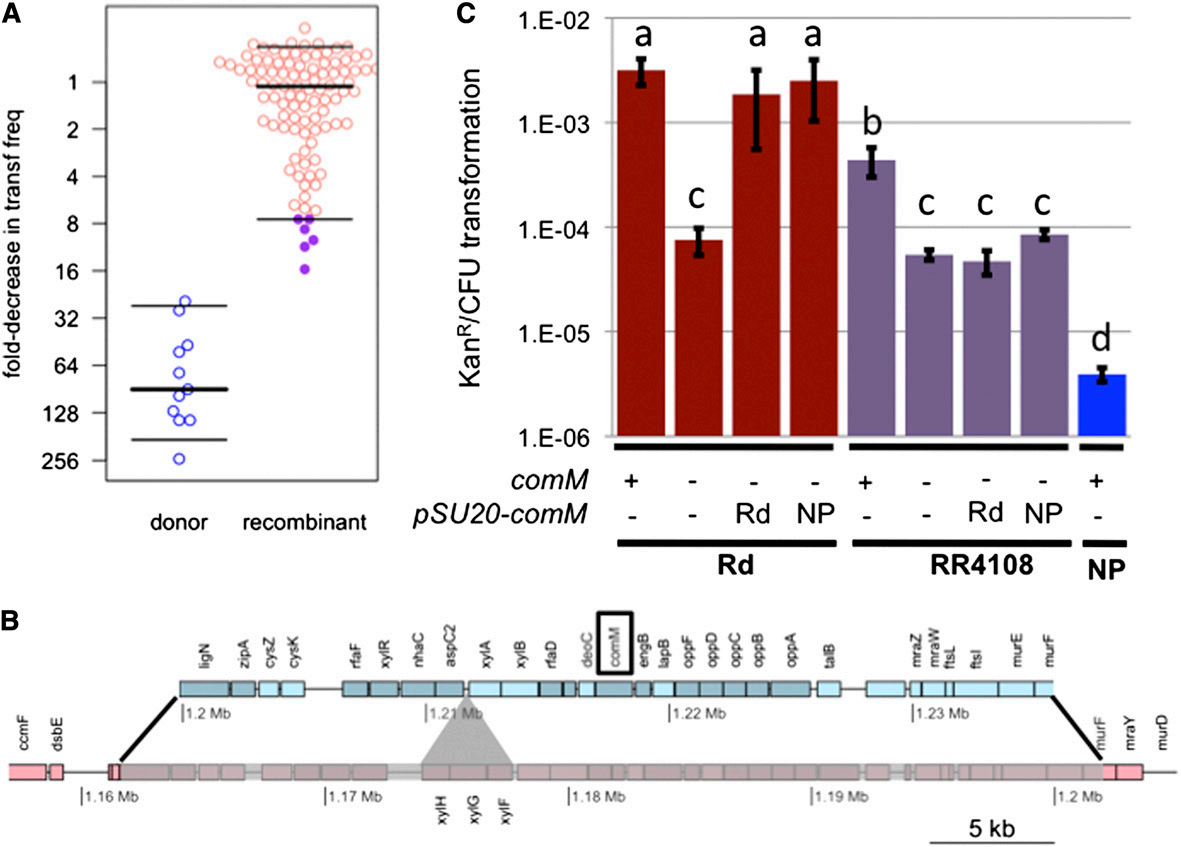
Transformation mapping of a transformability factor. (A) For the primary screen, “transformation-during growth” assays measure transformation frequencies (Kan^R^/CFU) of the set of 96 recombinants. Data are normalized to the frequencies observed for RR722 recipient controls in each experiment to account for experimental variation over the 11 independent assays performed. Thick lines indicate median value and thin lines indicate 5% and 95% quartiles. The RR3131 donor strain was included as a control. (B) Map of the donor segment interval in RR4108 is shown in blue replacing the syntenic genes in the recipient chromosome (pink). Black lines show donor segment endpoints. The recipient-specific xylose ABC transporter (shown with the gray triangle) was deleted in the recombinant. The *comM* gene is highlighted with a black box. (C) Transformation frequencies (Kan^R^/CFU) using the standard MIV method for the recipient and recombinant RR4108 strains with and without the *comM* gene and complementation by plasmids carrying either the donor or recipient alleles of the *comM* gene. The donor RR3131 (NP) strain is shown as a control. The *comM* deletion in RR3131 had a transformation frequency below the limit of detection. Bars show mean transformation frequencies and error bars show standard deviations from triplicate experiments. Letters above the bars group strains with no significant difference by paired *t*-test. Note that the difference between donor and recipient in the standard MIV assay is larger than in the primary screen.

Competence varies widely within species, but the underlying causes are unknown; understanding the genetic architecture of competence in natural populations could lead to a better understanding of the evolutionary forces that maintain it. For example, the 86-028NP donor strain has ~500-fold lower transformability than the Rd recipient and DNA uptake is ~20-fold lower (Maughan and Redfield 2009a,b). To assess the practicality of using natural transformation to map this variation, all 96 sequenced clones were tested for changes in transformability in a “transformation-during-growth” screen (Figure 10A), and the seven clones with the lowest transformation frequencies were re-assayed in MIV starvation medium (Figure S5A). This identified a single Nov^S^Nal^S^ recombinant clone (RR4108, from the unselected set, * in Figure 2C) with a reproducible 10-fold decrease in transformation (Figure 10B) (*t*-test *p*-value = 0.025). This recombinant has normal DNA uptake compared to Rd, so it must not have acquired alleles from the donor that affect either competence regulation or the outer membrane DNA uptake machinery: Following the method of (Sinha *et al.* 2012), assays using 50 ng of ^33^P-labeled 222 bp DNA fragment per milliliter of competent cells gave 26.0 ± 2.1% uptake by the recombinant RR4108, compared to 25.3 ± 3.4% uptake by Rd and 1.3 ± 0.4% uptake by 86-028NP.

The recombinant contains a single 40.3 kb recombination tract carrying 483 donor-specific SNVs, spanning ~30 genes and deleting a 3.7 kb xylose ABC transporter operon specific to the recipient strain (Figure 10B). At least one locus outside the RR4108 donor segment interval must be responsible for the remaining 50-fold difference in transformability between 86-028NP and Rd. Because 86-028NP has substantially reduced DNA uptake as well as transformability, some of this variation must affect either competence regulation or the uptake of DNA through the outer membrane. The normal transformation frequencies of the other 81 recombinants rule out major effects of ~1/3 of the donor-specific variation, including variation at 12 known competence genes. Subtle differences due to variation in these genes are possible, although normal MIV-induced transformation frequencies were seen for the six recombinants whose segments include any of the twelve (*t*-test *p*-value > 0.2 for all six; Figure S5B).

One known competence-regulated gene, *comM*, is found within the RR4108 donor segment. Null mutants of *comM* have a 42-fold defect in the Rd strain background (Figure 10C; *t*-test *p*-value = 0.028); its protein product acts after DNA uptake to promote recombination, consistent with the normal uptake observed in the RR4108 recombinant (Gwinn *et al.* 1998; Sinha *et al.* 2012). To test whether the two nonsynonymous differences distinguishing the *comM*-Rd and *comM*-NP alleles affect the gene’s activity, plasmids carrying each were used to test complementation of the deletion mutant’s phenotype. Both plasmids complemented the Rd *comMΔ*::*spc* mutant equally well (Figure 10C; *t*-test *p*-value = 0.118), indicating nonsynonymous *comM* differences are not responsible for the phenotypic change.

Additional experiments complicated matters; these indicate that although genetic variation within *comM* is not responsible for the altered phenotype, the chromosomal *comM* locus is required for the difference between the strains to be observed: First, deleting *comM* from RR4108 reduced transformation to the same levels as in Rd (Figure 10C; *t*-test *p*-value = 0.32). This might suggest that ComM activity contributes more to transformability in the recipient background, but the requirement for ComM was substantially stronger in the 86-028NP donor background (wild type: 3.9 ± 0.6 × 10^−6^ Kan^R^/CFU; *comM* deletion: <7.9 ± 0.2 × 10^−9^). Second, the plasmids carrying the Rd and NP alleles of *comM* failed to complement a RR4108 *comMΔ*::*spc* mutant (Figure 10C; *t*-test *p*-values > 0.05). This might indicate that variation in the RR4108 donor segment modulates *comM* activity *in cis*, but both promoter and terminator are invariant between the strains and the plasmid insert carrying the *comM* alleles was flanked by ±700 bp.

In summary, this pilot mapping experiment has used natural transformation to identify a genetic interval carrying natural variation that affects transformability itself. We found that the variation in transformability is a polygenic trait with differences affecting both pre- and postuptake steps, and we have begun unraveling complex interactions between the *comM* locus and unidentified but linked natural variation.

## Discussion

### Experimental genomics and bacterial genetic recombination

Although the evolutionarily selected function of natural competence remains controversial—since DNA can provide nutrients as well as genetic information—the consequences of transformational recombination are not in doubt (Didelot and Maiden 2010; Feil and Spratt 2001; Moradigaravand and Engelstadter 2013; Redfield 2001). In this study, we examined the genome-wide recombination that arose when competent *H. influenzae* cells were incubated with DNA from a diverged clinical isolate, finding that the average cell’s daughters replaced ~1% of their chromosomes in multiple independent recombination events that spanned many genes. These data will help constrain parameters used for genome-wide inferences of historical recombination in natural populations, for example by providing a rate parameter to describe the exponential distribution of donor segment lengths (Figure 3C) (Didelot *et al.* 2010). Comparisons of such parameter estimates with those from genome-wide studies of other species or other horizontal gene transfer processes will help disentangle how modulations in the mechanism of genetic recombination affect its long-term genomic consequences (Croucher *et al.* 2012; Feil and Spratt 2001; Gray *et al.* 2013; Wilson 2012).

### Comparison of natural transformation in *H. influenzae* and *S. pneumoniae*

Although the species are very distantly related, our genome-wide analyses of transformation in *H. influenzae* reached conclusions similar to those for the Gram-positive bacterium *S. pneumoniae* (Croucher *et al.* 2012; Mell *et al.* 2011): (a) Competent cells typically take up and recombine multiple donor molecules; (b) recombination tracts can span dozens of genes and hundreds of genetic variants; (c) tracts are often mosaic clusters of donor segments; (d) nucleotide divergence has surprisingly little effect on the locations of recombinant segments; (e) SVs transform at lower rates than SNVs; (f) deletions may transform more easily than insertions; and (g) no strong transformation hotspots were identified. Herein, we contrast the genome-wide analyses of transformation in *H. influenzae* and *S. pneumoniae*, but it is worth noting that results from the two studies might not reflect species-wide recombination parameters, as we still know very little about how such parameters vary within species or for different combinations of donor and recipient genomes.

Donor segments in *S. pneumoniae* are shorter than in *H. influenzae* (mean size 2.3 *vs.* 6.9 kb), which could reflect their processing by distinct DNA-uptake mechanisms. Although the Gram-negative *H. influenzae* translocates large donor molecules from the periplasm through its inner membrane, *S. pneumoniae* and other Gram-positive bacteria process dsDNAs with membrane-bound nucleases before translocation through their single cell membrane (Lacks and Neuberger 1975). The mosaic recombination tracts seen in both species likely only sometimes result from heteroduplex correction mechanisms and instead could often be due to endonuclease activities acting prior to strand exchange, or prior to translocation into the cytoplasm.

Although transformation hotspots were not identified in either species, the power to detect variation in transformation frequency across the genome was limited in both cases, and genetic experiments have found that transformation rates are affected by nucleotide divergence and can independently vary between loci (Lawrence 2002; Mell *et al.* 2011; Ray *et al.* 2009). Our observation in *H. influenzae* that transformation decreases irregularly around the selected antibiotic resistance loci (Figure 5) contrasts with *S. pneumoniae* and may indicate higher heterogeneity in transformation frequencies.

Accurate quantification of genome-wide variation in transformation frequency will require either sequencing much larger sets of recombinants or deeply sequencing large pools of recombinant genomes. These experiments could more precisely tease apart the effects of divergence and such locus-specific factors as proximity to the origin of DNA replication or the effects of chromosome structural proteins (Ray *et al.* 2009). Estimating these effects will be particularly crucial for population genetic inferences, for example distinguishing between natural selection at a locus favoring recombination and simply higher recombination rates at that locus.

### Heteroduplex segregation and correction

Our observation of mixed donor segments clustered around the predicted origin of replication is consistent with heteroduplex segregation, where recombination events occurred after DNA replication of the unselected locus but before replication of the selected locus (Figure 1C). Consideration of heteroduplex segregation also led to our interpretation that the excess of very short donor segments (Figure 3D) could be the result of donor-directed heteroduplex correction from recombined donor DNA that was subsequently lost by segregation. Confirming that mismatch repair mutants lack these very short segments would have implications for population-level analyses of recombination, since such methods typically identify putative recombination events by their inclusion of many nearby polymorphic sites.

### Proof-of-principle genetic mapping of natural variation in transformability

Our pilot mapping experiment shows that plummeting sequencing costs make natural transformation combined with genomics a powerful means to map natural phenotypic variation. Recombinants generated by natural transformation are equivalent to the “nearly isogenic lines” used for genetic mapping in eukaryotes, though in bacteria they are considerably easier to generate and cheaper to comprehensively genotype. This allowed us to use our small mapping population to rapidly identify an interval (comprising ~2% of the genome) that contained natural variation with a 10-fold effect on transformability. To our knowledge, this is the first time transformation has been used to identify a bacterial quantitative trait locus, and importantly the causal variation is not accounted for by any of the known competence genes (Sinha *et al.* 2012). Extending this transformation genomics approach to other phenotypes will be particularly powerful when the trait conferred by donor DNA is selectable, as this will obviate the need to screen for phenotypic changes in large recombinant mapping populations. Many such traits are important to pathogenesis and vary widely between clinical isolates of *H. influenzae*; these include antibiotic resistances, resistance to human complement-mediated killing, and intracellular invasion of airway epithelial cells (Morey *et al.* 2011; Tristram *et al.* 2007; Williams *et al.* 2001).

## Supplementary Material

Supporting Information
