# Supplementary material for: Extensive Cotransformation of Natural Variation into Chromosomes of Naturally Competent Haemophilus influenzae
Source: G3 (Bethesda). 2014 Feb 25;4(4):717–31. doi: 10.1534/g3.113.009597 (PMC4059242; doi:10.1534/g3.113.009597)
Supplement: Supporting Information [file supp_4_4_717__index.html]

Extensive Cotransformation of Natural Variation into Chromosomes of Naturally Competent Haemophilus influenzae — Supporting Information 

# Extensive Cotransformation of Natural Variation into Chromosomes of Naturally Competent *Haemophilus influenzae*

## Supporting Information for Mell *et al.*, 2014

**Files in this Data Supplement:**

- Supporting Information - Figures S1-S5, Table S1, File S1, and description of Files S2-S3 (PDF, 1 MB)
- Figure S1 - Summary of read alignments to the two references. (PDF, 458 KB)
- Figure S2 - Post-alignment read depth is highly variable but consistent between samples. (PDF, 593 KB)
- Figure S3 - Spurious clustering of two segments in RR4049. (PDF, 517 KB)
- Figure S4 - Spurious independence of adjacent segments in RR4036. (PDF, 509 KB)
- Figure S5 - Quantitative retesting of transformability in recombinant clones. (PDF, 459 KB)
- Table S1 - Primers Used (PDF, 147 KB)
- File S1 - Supplementary Methods (PDF, 163 KB)
- File S2 - Genomic DNA samples sequenced (.zip, 2 KB)
- File S3 - Donor segments detected (.zip, 10 KB)
