# Supplementary material for: Extensive Cotransformation of Natural Variation into Chromosomes of Naturally Competent Haemophilus influenzae
Source: G3 (Bethesda). 2014 Feb 25;4(4):717–31. doi: 10.1534/g3.113.009597 (PMC4059242; doi:10.1534/g3.113.009597)
Supplement: Supporting Information [file supp_g3.113.009597_009597SI.pdf]

G3 · Genes | Genomes | Genetics

EXTENSIVE CO-TRANSFORMATION OF NATURAL VARIATION INTO CHROMOSOMES OF NATURALLY COMPETENT *HAEMOPHILUS INFLUENZAE*

Joshua Chang Mell*[1], Jae Yun Lee§, Marlo Firme§, Sunita Sinha†, and Rosemary J. Redfield*

University of British Columbia, Vancouver, BC V6T 1Z3, Canada, * Department of Zoology, § Genome Sciences and Technology Graduate Program, † Department of Pharmacy Sciences

[1] Corresponding author: University of British Columbia, Dept. of Zoology, Life Sciences Institute, 2350 Health Sciences Mall, Vancouver, BC V6T 1Z3, Canada. Email: mell@zoology.ubc.ca

NCBI short-read archive project accession number: SRP036875

DOI: 10.1534/g3.113.009597

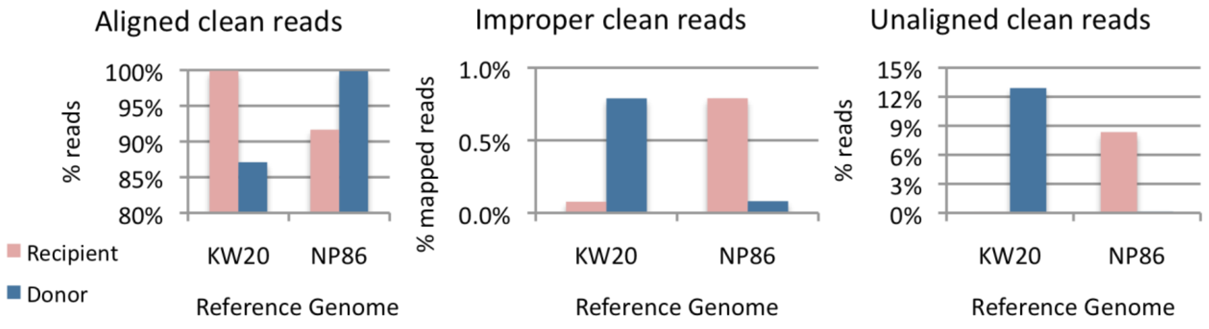

Figure S1 Summary of read alignments to the two references. Percent of control reads post-adaptor trimming that: (**A**) mapped with proper pairing, (**B**) mapped with improper pairing, and (**C**) remained unmapped.

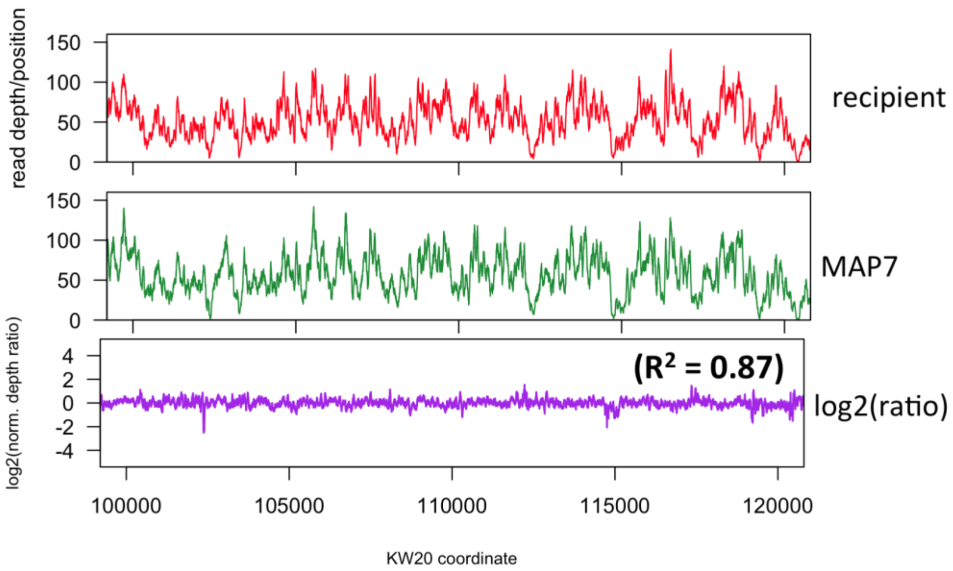

Figure S2 Post-alignment read depth is highly variable but consistent between samples. Example shown for 20 kb interval comparing read depth at each position for the recipient and MAP7 control strains (top and middle panels). The bottom panel shows the log2(ratio of MAP7/recipient read depths). The correlation was high across the whole genome (R^2=0.87).

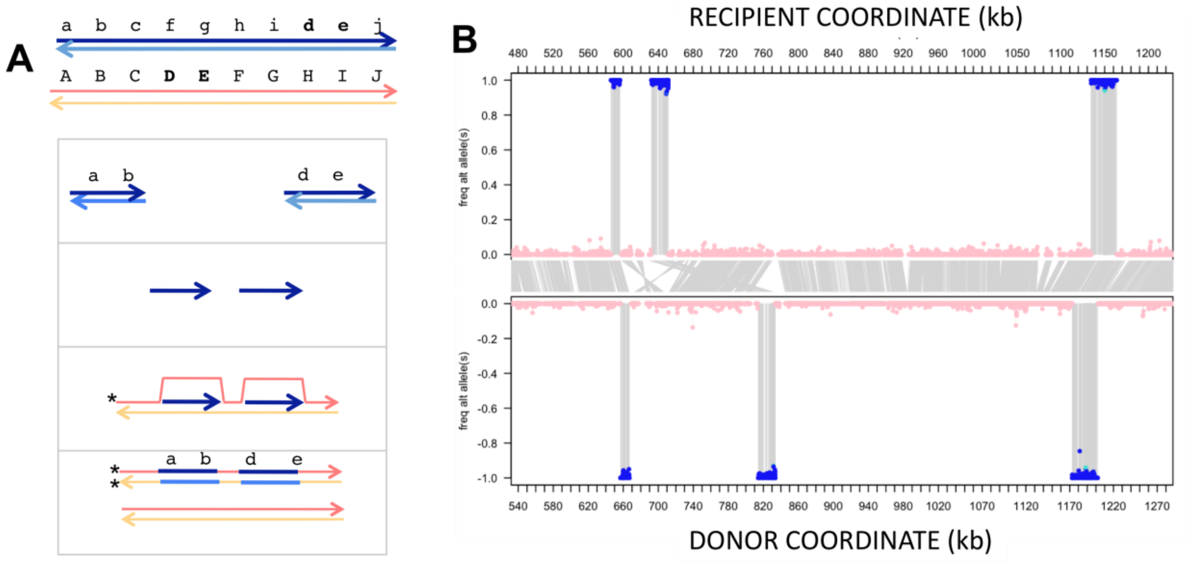

Figure S3 Spurious clustering of two segments in RR4049. (**A**) Schematic of inferred transformation intermediates. (**B**) Plot of donor-specific allele frequency against the recipient genome (top panel) and the donor genome (bottom panel); connecting lines between the plots show all syntenic SNV positions connecting the two references. The two donor segments in the top left appear to be ~50 kb apart but were derived from segments ~150 kb apart. A very similar pattern was seen for RR4050.

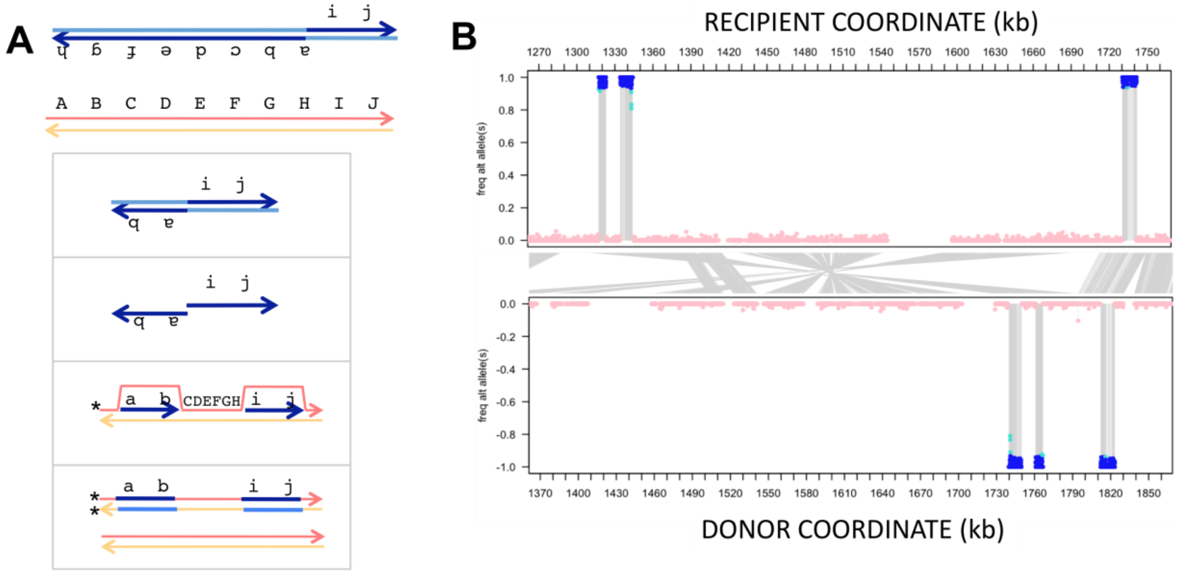

Figure S4 Spurious independence of adjacent segments in RR4036. Plots as in Figure S3. The right-hand segment appears to be >450 kb from the other two on the recipient genome, but all three segments span <100 kb of the donor genome. If the three donor segments were derived from a single molecule, double-translocation (translocation from both ends) would be required to give the observed pattern.

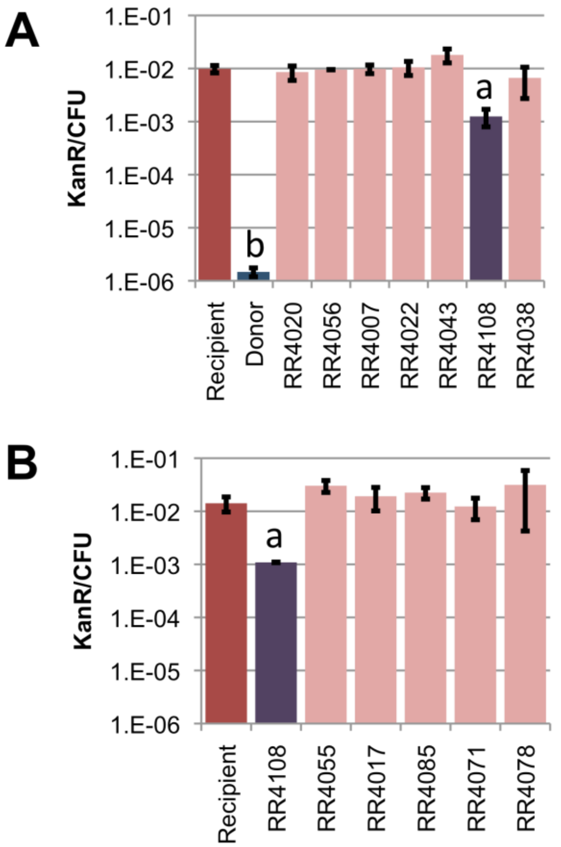

Figure S5 Quantitative retesting of transformability in recombinant clones. Values Kan^R/CFU transformation frequencies for MIV starvation cultures and represent the mean of triplicate experiments and error bars show ± standard deviation. Letters indicate significant differences from the Rd recipient strain by paired t-test. (**A**) Retest of the seven recombinants that had shown a reduced transformation frequency in the primary 'transformation-during-growth' assay (purple dots in **Figure 10A**). (**B**) Retest of the six recombinants for which donor segments spanned all or part of a known competence genes. Genes spanned by donor segments are as follows: RR4108 spanned *comM*; RR4055 spanned *sxy* and *rec1*; RR4017 spanned part of *pilA*; RR4085 spanned part of *pilB* and all of *pilCD*; RR4071 spanned *comNOPQ* and *radC*; and RR4078 spanned *ligA*. Known competence-regulated genes not spanned by donor segments were: *comABCDEF*, *comE1*, *pilF2*, *rec2*, and the unknowns *HI0659, HI0660, HI1631,* and *HI0365.*

Table S1 Primers Used

Name	Purpose	Sequence	Rd-start	Rd-stop	NP-start	NP-stop
F_Rd_A.E10	test tract 106	**C**GTTTCTTTATTCTTATTTGG**T**GC	805,155	805,178	*871,522*	*871,545*
R_Rd_A.E10	test tract 106	GAAAGTACAAATAG**A**GC**G**AA	805,496	805,477	*871,863*	*871,844*
F_NP_A.E10	test tract 106	**T**GTTTCTTTATTCTTATTTGG**A**GC	805,155	805,178	*871,522*	*871,545*
R_NP_A.E10	test tract 106	GAAAGTACAAATAG**C**GC**A**AA	805,496	805,477	*871,863*	*871,844*
F_Rd_B.E10	test tract 106	AAG**C**CG**C**AAAGAATTACAAGA	831,046	831,066	*896,435*	*896,455*
R_Rd_B.E10	test tract 106	TGG**C**GCAGTTTC**A**TCATA	831,517	831,500	*896,906*	*896,889*
F_NP_B.E10	test tract 106	AAG**T**CG**T**AAAGAATTACAAGA	831,046	831,066	*896,435*	*896,455*
R_NP_B.E10	test tract 106	TGG**T**GCAGTTTC**G**TCATA	831,517	831,500	*896,906*	*896,889*
F_Rd_comM	test tract 143	GGCGGAA**GTG**TTCGCTGCCT	1,181,122	1,181,141	*1,214,974*	*1,214,993*
F_NP_comM	test tract 143	GGCGGAA**TCC**TTCGCTGCCT	1,181,122	1,181,141	*1,214,974*	*1,214,993*
R-comM	test tract 143	GCAGGGCGTAAACCGTGGCAT	1,186,049	1,186,030	*1,219,901*	*1,219,881*
F-murE-Rd	test tract 143	ACGC**G**CAATGGTGGAAGGCTAC	1,199,339	1,199,360	*1,233,187*	*1,233,208*
F-murE-NP	test tract 143	ACGC**A**CAATGGTGGAAGGCTAC	1,199,339	1,199,360	*1,233,187*	*1,233,208*
R-murE	test tract 143	AACAAGCGCAAGCGCACCCT	1,201,362	1,201,343	*1,235,210*	*1,235,191*
F-xylFlank	test tract 143	AGTCTGCGTGGACATCATCGGA	1,173,109	1,173,130	*1,210,948*	*1,210,969*
R-xylFlank	test tract 143	CCAGCAAGCGTCGCGTGGTTA	1,178,735	1,178,715	*1,212,587*	*1,212,567*
F-xylG-Rd	test tract 143	TGCCGTGTTTCTTCCGATCTTCAGG	1,175,579	1,175,603	*0*	*0*
R-xylG-Rd	test tract 143	ACAGCAGGTGCAATTAGATGCCG	1,176,249	1,176,227	*0*	*0*
*comM*_F	*comMΔ::spc*	GCGGTAAGTTTTTCGGGTAA	1,182,091	1,182,111	NA	NA
*comM*_R	*comMΔ::spc*	CCACACCAAGTTCCCCAAAA	1,185,779	1,185,799	NA	NA
*comM*_*Eco*RI_F	*pSU20-comM*	G**GAATTC**CCTGAAGATACGGGCATTG	1,182,399	1,182,418	NA	NA
*comM*_*Eco*RI_R	*pSU20-comM*	G**GAATTC**GGAGGAAAATAATGACATCAAAT	1,185,284	1,185,307	NA	NA

For test primers, bold underlined bases indicate variants between the recipient and donor genomes (RR722 and RR3131). Genome coordinates of primer sequences are provided for both genomes. For cloning primers, bold underlined GAATTC indicates the *Eco*RI restriction site.

File S1

Supplementary Methods

DNA sequencing, data processing, and short-read alignments: DNA samples were submitted to the Michael Smith Genome Sciences Centre (Vancouver, BC, Canada) for multiplexed Illumina library construction, and the resulting libraries were sequenced in pools of 20-24 per lane on an Illumina HiSeq instrument, yielding ~30 million paired-end reads of 50 nt per end from each lane. Data was provided by the sequencing centre as unmapped BAM files, divided into 91 files based on the samples' multiplexing barcodes.

Datasets were first purged of read pairs failing quality checks. Paired reads that failed the Illumina chastity filter were removed with samtools v0.1.18 (samtools view -F 0x200) (LI *et al.* 2009). The resulting BAM files were then converted to FastQ files using samtools view and the Unix awk utility. The utility sortPairedReads (https://github.com/tanghaibao/trimReads) was used on the resulting FastQs with default settings to set aside read pairs contaminated with Illumina adapter sequences and/or strings of low-quality bases. (The FastA file used for specifying the adaptor sequences included reverse complements.)

The filtered FastQs were then aligned to both donor and recipient reference sequences (Rd KW20 and 86-028NP, GenBank accessions L42023.1 and CP000057.2, respectively) with the BWA short-read aligner (LI and DURBIN 2010)(aln settings: -Y -l 20 -n 8 -o 3 -e 3; sampe used defaults). Mapped sorted BAM files were produced using samtools (view | sort | rmdup). Finally, the GATK IndelRealigner v1.4 was used with default settings to multiply align reads that mapped with short indels and thus minimize alignment artifacts at and near these positions (DEPRISTO *et al.* 2011; MCKENNA *et al.* 2010). Read quality and alignment statistics were collected with FastQC (http://www.bioinformatics.babraham.ac.uk/projects/fastqc/) and samtools flagstat. Read depths and intersects with specific genomic intervals were determined with BedTools v2.16 (see below)(QUINLAN and HALL 2010).

Whole-genome alignment: Alignment of the donor and recipient reference sequences used progressiveMauve v2.3.1 (DARLING *et al.* 2010) with default settings. The resulting XMFA alignments were parsed with a custom R script to generate 'lift-over' tables, which cross-referenced the genome coordinates between the two reference sequences. Because Mauve whole-genome alignment gave slightly different results depending on the order the reference sequences were entered, lift-overs using both reference orders were calculated.

Genotype calling—SNVs: Single-nucleotide variants (SNVs) and short indels (<10 bp) were identified using samtools (samtools mpileup -Eug) with all 91 BAM alignments simultaneously, followed by genotype calling and variant filtering (bcftools view -vg). The two resulting BCF files contained a line for each genomic position where ≥1 sample had a non-reference (alternate) genotype. Because samtools/bcftools were designed to work with diploid genomes, 'heterozygous' or 'mixed' genotypes were called when aligned reads supported both the reference and alternate alleles. Variant call data against both references was deposited as BCFs

Manual inspection of BAM and BCF files used the Integrative Genomics Viewer (IGV) v2.1.17 (THORVALDSDOTTIR *et al.* 2013) to evaluate each filtering step, to validate recombination breakpoints, and to investigate 'mixed' recombination tracts (consisting of contiguous runs of "heterozygous" donor-specific variants). To independently derive allele frequencies at each position, a perl script (https://github.com/riverlee/pileup2base) was used to count bases directly from the pileup output of individual samples.

The two BCF files (corresponding to variant calls against each reference) were decompressed (bcftools view) and parsed (awk) to simplify the output for analysis in R, retaining for each line: the genomic coordinate, the reference allele, the alternate allele(s), and the genotype called for each of the 91 samples (REF for 0/0 reference calls, ALT for 1/1 alternate calls, and MIX for 'heterozygous' 0/1 calls). At the four known MAP7-specific variants in donor strain RR3131 (MELL *et al.* 2011), the reference alleles, alternate alleles, and genotype calls were manually corrected, so that they would correctly identify these donor-specific alleles and escape the stringent filtering described below. To identify a set of 'gold-standard' SNV positions that reliably distinguished donor from recipient, a set of filters were applied to the two genotype files (one corresponding to each reference sequence), as follows and summarized in **TABLE 3**:

(1) The position had a consistent non-zero 'lift-over' to the other genome, as determined by whole-genome alignment. This eliminated many variant calls due to ambiguous alignments around indels and at repetitive sequences. It also eliminated short indel variants, which were handled separately (see below).

(2) Control reads from the donor and recipient strains unambiguously distinguished donor from recipient alleles in the expected fashion, *i.e.* (a) donor reads supported the alternate allele when aligned to the recipient but supported the reference when aligned to the donor, and also (b) recipient reads supported the alternate allele when aligned to the donor but supported the reference when aligned to the recipient. This step recoded the genotypes as 'recipient', 'donor', or 'mixed', and it eliminated variants arising due to differences between the parental strain genomes and their respective references, as well as those with

‘mixed’ genotype calls in the parental controls. This filter also excluded ‘novel’ alleles differing from either parental control, so potentially excluded true mutations that arose in the recombinant clones (see below).

(3) The total fraction of ‘mixed’ genotypes across all 91 sequences was less than 5% (*i.e.* no more than 4 clones had a mixed genotype at a particular position). This filter eliminated error-prone positions resulting from systematic sequencing and alignment errors (particularly for repetitive sequences).

(4) Finally, the ‘gold-standard’ set of variants was reduced to those that passed all three filters, not only for the individual genotype file, but also whose corresponding lift-over position passed all three filters in the reciprocal genotype file.

Genotype calling—SVs: To classify structural variants (SVs), we used a three-step approach, summarized in **Table 4**. First, large indels, inversions, and other rearrangements that distinguish the donor and recipient genomes were identified from the .backbone and “Export gaps…” outputs of progressiveMauve, and the coordinates of each SV were collected for each reference genome. The genomic coordinates of unaligned DNA segments were determined from their flanking aligned breakpoints, and the total set of SV intervals were converted into BED files. Second, the ability of short-read paired-end sequencing to identify these SVs relied on the fact that reads should map to SV breakpoints without gaps or clipping when they are being aligned to the correct genome, but none should map when aligned to the incorrect genome. From each alignment, ungapped unclipped read alignments were extracted at each breakpoint, requiring 5 flanking bases on either side by applying awk and intersectBed. Read depth statistics at each SV breakpoint were extracted using coverageBed. Third, a “gold-standard” set of SVs was determined as those whose breakpoints had high depth when recipient reads were aligned to the recipient genome but low depth against the donor genome and *vice versa*, as determined by Fisher’s exact test (*p-value* < 0.001 per marker). For larger indels (>250 bp) read depth statistics were also extracted from each alignment to confirm the presence/absence of accessory loci. The genotype of each SV for each recombinant was then determined with two Fisher’s exact tests, one against each reference, yielding four potential genotypes: recipient, donor, both, or neither. As with SNVs, SV markers for which >4 samples were both or neither were excluded, since the genotyping method was unreliable at these markers.

Identifying and defining donor segments and breakpoint intervals: Since donor DNAs are known to transform the *H. influenzae* chromosome as relatively long ssDNA molecules, ‘donor segments’ were defined as contiguous runs of gold-standard donor-specific SNVs (including those positions with mixed donor/recipient alleles). Donor segments were called from each genotype file (corresponding to each reference sequence). Breakpoint intervals were initially defined by the coordinate of each donor segments’ outermost donor-specific variants and their nearest adjacent recipient-specific variants. Cross-validation of donor segments in each of the two sets then required that all four breakpoint-defining coordinates in one reference lifted over uniquely to coordinates defining a segment in the other. This cross-validation eliminated most of the putative donor segments with a length of only 1, especially those with mixed genotypes, since many of these arose due to alignment artifacts, rather than representing true transformation events.

Because the “gold-standard” set of SNVs initially excluded indels and other SVs, as well as artifact-prone variants near these and in repetitive DNA, the breakpoint intervals were further refined by interpolation of the SV genotypes. Transforming SVs found within these donor segments were identified as described above and validated by inspection in IGV. Because some DNA samples were mixtures of more than one clone (or the clone was otherwise a mixture of genotypes), contiguous runs of mixed donor/recipient SNVs were deemed ‘mixed’ donor segments. Singleton positions with a mixed genotype (either within or outside donor segments) were manually examined in IGV, and the genotypes were manually adjusted when the donor-specific allele frequency was >90% or <10% (from “mixed” to “donor” or “recipient”, respectively).

References in Supplementary Methods

Darling, A. E., B. Mau and N. T. Perna, 2010 progressiveMauve: multiple genome alignment with gene gain, loss and rearrangement. PLoS One **5:** e11147.

DePristo, M. A., E. Banks, R. Poplin, K. V. Garimella, J. R. Maguire *et al.*, 2011 A framework for variation discovery and genotyping using next-generation DNA sequencing data. Nat Genet **43:** 491-498.

Li, H., and R. Durbin, 2010 Fast and accurate long read alignment with Burrows-Wheeler transform. Bioinformatics.

Li, H., B. Handsaker, A. Wysoker, T. Fennell, J. Ruan *et al.*, 2009 The Sequence Alignment/Map format and SAMtools. Bioinformatics **25:** 2078-2079.

McKenna, A., M. Hanna, E. Banks, A. Sivachenko, K. Cibulskis *et al.*, 2010 The Genome Analysis Toolkit: a MapReduce framework for analyzing next-generation DNA sequencing data. Genome Res **20:** 1297-1303.

Mell, J. C., S. Shumilina, I. M. Hall and R. J. Redfield, 2011 Transformation of natural genetic variation into Haemophilus influenzae genomes. PLoS Pathog **7:** e1002151.

Quinlan, A. R., and I. M. Hall, 2010 BEDTools: a flexible suite of utilities for comparing genomic features. Bioinformatics **26:** 841-842.

Thorvaldsdottir, H., J. T. Robinson and J. P. Mesirov, 2013 Integrative Genomics Viewer (IGV): high-performance genomics data visualization and exploration. Brief Bioinform **14:** 178-192.

File S2

Genomic DNA samples sequenced

File S2 is a tab-delimited text file (with header) that reports metadata on each DNA sample sequenced (indicated in **TABLE 1**), available for download at http://www.g3journal.org/lookup/suppl/doi:10.1534/g3.113.009597/-/DC1. Well IDs are used in the naming of the BAM files deposited at the NCBI short-read archive under project accession SRP036875.

Explanation of columns is below:

Well: Coordinate in 96-well plate during sequencing library construction

Sample: Strain ID(s) of stored culture grown from a single colony. For samples G01-G08, pairs of strains were pooled.

Selection: Indicates presence/absence of the Nov^R and Nal^R markers. NovR and NalR indicate the single resistances; NovRNalR indicates double; and None indicates sensitivity to both antibiotics.

Experiment: Source of cloned colony. MIV1-MIV3 indicate that colonies were picked from transformations using the standard competent protocol; LATE1 indicates that colonies were picked from a late-log transformation. RECIP, CONTROL, and DONOR refer to the recipient Rd strain (RR722), the MAP7 control strain (RR666), and the donor strain derived from 86-028NP (RR3131).

File S3

Donor segments detected

File S3 is a tab-delimited text file (with header) of all detected donor segments, available for download at http://www.g3journal.org/lookup/suppl/doi:10.1534/g3.113.009597/-/DC1. Samples with no recombinants detected, as well as the parental strains, have no corresponding rows in this file.

Explanation of columns is below:

Well: Coordinate in 96-well plate during sequencing library construction

Sample: Strain ID(s) of stored culture grown from a single colony. For samples G01-G08, pairs of strains were pooled.

Seg: ID for each donor segment

Trct: ID for each recombination tract (segments clustered within 100 kb). Ties for tract endpoints within a clone were broken randomly.

Type: SEL=spanning selected Nov^R or Nal^R resistance allele; ADJ=in same tract as selected segment; IND=>100 kb from selected allele

MinKB: Minimum size of recombination tract in kilobases based on outermost donor-specific variants of each segment

SNVs: Count of single-nucleotide variants detected in the segment

Dsnv: Count of SNVs for which the genotype was unambiguously from the donor

Msnv: Count of SNVs determined to be "heterozygous" or with reads supporting both donor and recipient alleles

SVs: Count of structural variants in segment

Deleted: Total number of base pairs deleted by SVs in segment

Inserted: Total number of base pairs inserted by SVs in segment

LeftSV: Is an SV the nearest flanking recipient allele on the left of the segment?

LeftR-Rd: Coordinate in the Rd genome of the recipient allele nearest the left edge of the segment

LeftD-Rd: Coordinate in the Rd genome of the leftmost donor-specific allele in the segment

RghtD-Rd: Coordinate in the Rd genome of the rightmost donor-specific allele in the segment

RghtR-Rd: Coordinate in the Rd genome of the recipient allele nearest the right edge of the segment

RghtSV: Is an SV the nearest recipient allele on the right of the segment?

Invert: Is the donor segment inverted in the 86-028NP genome?

LeftR-NP: Coordinate in the 86-028NP genome of the recipient allele nearest the left edge of the segment

LeftD-NP: Coordinate in the 86-028NP genome of the leftmost donor-specific allele in the segment

RghtD-NP: Coordinate in the 86-028NP genome of the rightmost donor-specific allele in the segment

RghtR-NP: Coordinate in the 86-028NP genome of the recipient allele nearest the right edge of the segment
