# Supplementary material for: Extensive Cotransformation of Natural Variation into Chromosomes of Naturally Competent Haemophilus influenzae
Source: G3 (Bethesda). 2014 Feb 25;4(4):717–31. doi: 10.1534/g3.113.009597 (PMC4059242; doi:10.1534/g3.113.009597)
Supplement: Supporting Information [file supp_g3.113.009597_FigureS1.pdf]

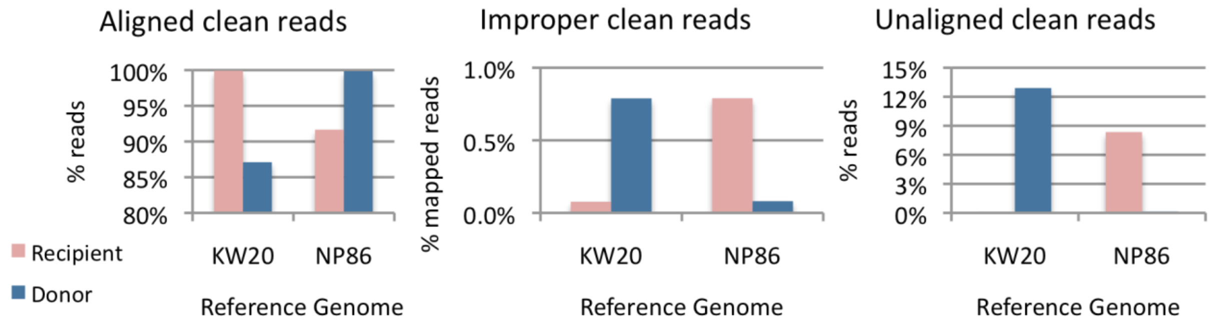

Figure S1 Summary of read alignments to the two references. Percent of control reads post-adaptor trimming that: (**A**) mapped with proper pairing, (**B**) mapped with improper pairing, and (**C**) remained unmapped.
