# Supplementary material for: Extensive Cotransformation of Natural Variation into Chromosomes of Naturally Competent Haemophilus influenzae
Source: G3 (Bethesda). 2014 Feb 25;4(4):717–31. doi: 10.1534/g3.113.009597 (PMC4059242; doi:10.1534/g3.113.009597)
Supplement: Supporting Information [file supp_g3.113.009597_FigureS2.pdf]

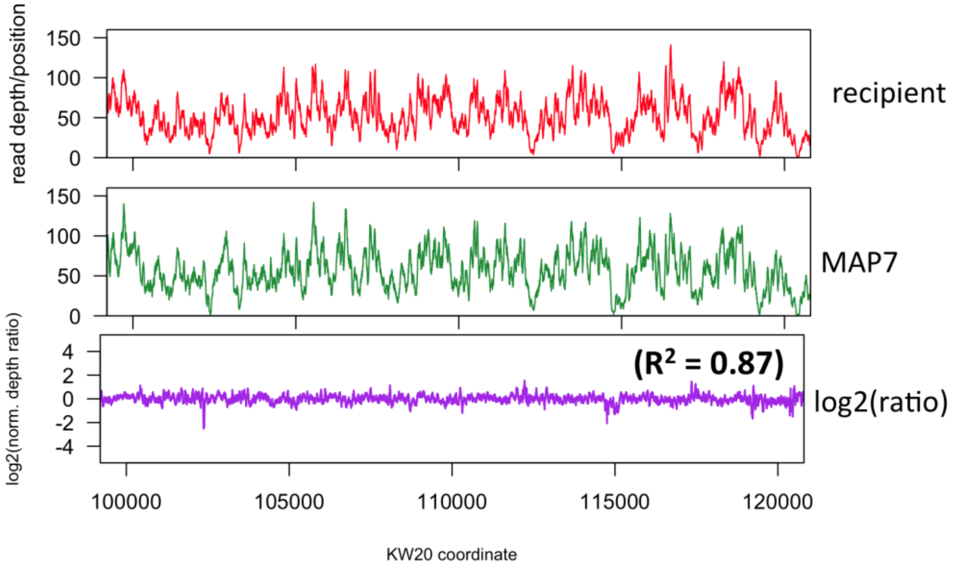

Figure S2 Post-alignment read depth is highly variable but consistent between samples. Example shown for 20 kb interval comparing read depth at each position for the recipient and MAP7 control strains (top and middle panels). The bottom panel shows the log2(ratio of MAP7/recipient read depths). The correlation was high across the whole genome (R^2=0.87).
