# Supplementary material for: Extensive Cotransformation of Natural Variation into Chromosomes of Naturally Competent Haemophilus influenzae
Source: G3 (Bethesda). 2014 Feb 25;4(4):717–31. doi: 10.1534/g3.113.009597 (PMC4059242; doi:10.1534/g3.113.009597)
Supplement: Supporting Information [file supp_g3.113.009597_FigureS3.pdf]

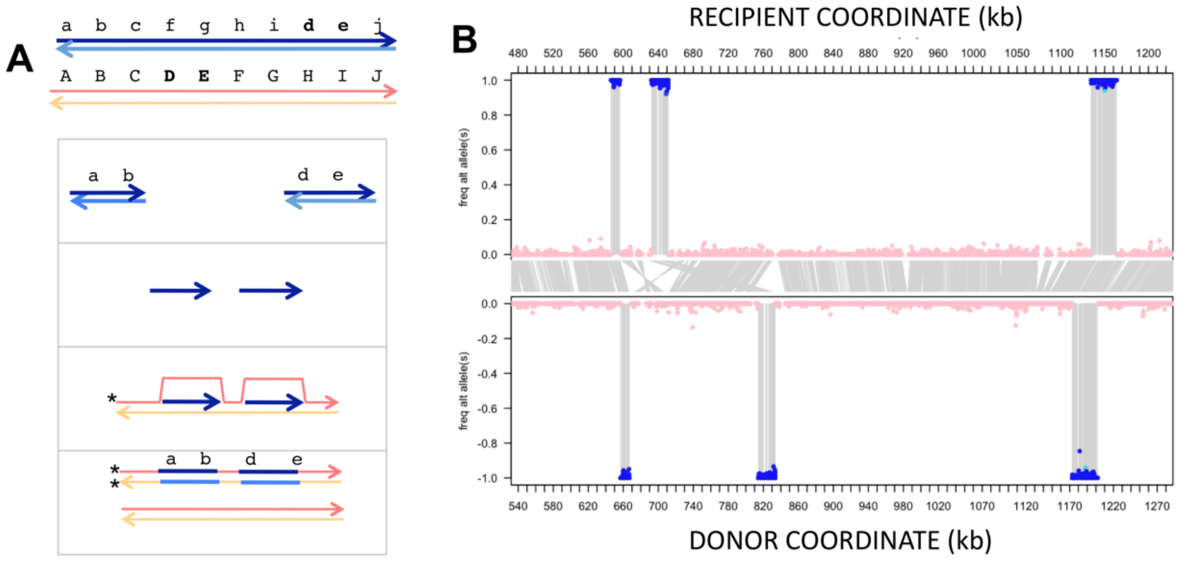

Figure S3 Spurious clustering of two segments in RR4049. (**A**) Schematic of inferred transformation intermediates. (**B**) Plot of donor-specific allele frequency against the recipient genome (top panel) and the donor genome (bottom panel); connecting lines between the plots show all syntenic SNV positions connecting the two references. The two donor segments in the top left appear to be ~50 kb apart but were derived from segments ~150 kb apart. A very similar pattern was seen for RR4050.
