# Supplementary material for: Extensive Cotransformation of Natural Variation into Chromosomes of Naturally Competent Haemophilus influenzae
Source: G3 (Bethesda). 2014 Feb 25;4(4):717–31. doi: 10.1534/g3.113.009597 (PMC4059242; doi:10.1534/g3.113.009597)
Supplement: Supporting Information [file supp_g3.113.009597_FigureS4.pdf]

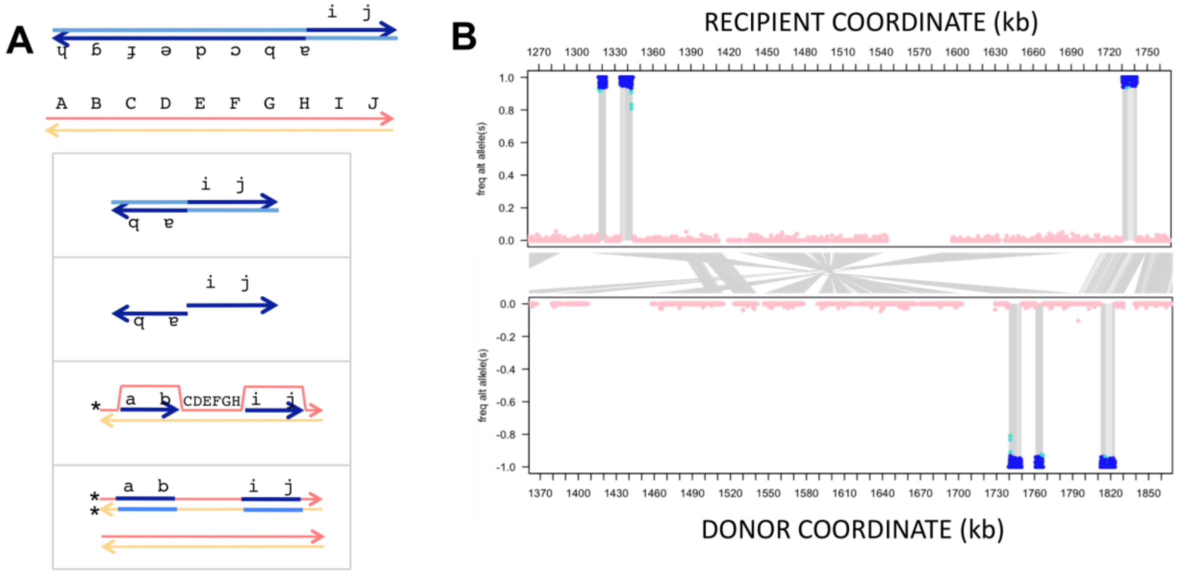

Figure S4 Spurious independence of adjacent segments in RR4036. Plots as in Figure S3. The right-hand segment appears to be >450 kb from the other two on the recipient genome, but all three segments span <100 kb of the donor genome. If the three donor segments were derived from a single molecule, double-translocation (translocation from both ends) would be required to give the observed pattern.
