# Supplementary material for: Extensive Cotransformation of Natural Variation into Chromosomes of Naturally Competent Haemophilus influenzae
Source: G3 (Bethesda). 2014 Feb 25;4(4):717–31. doi: 10.1534/g3.113.009597 (PMC4059242; doi:10.1534/g3.113.009597)
Supplement: Supporting Information [file supp_g3.113.009597_FigureS5.pdf]

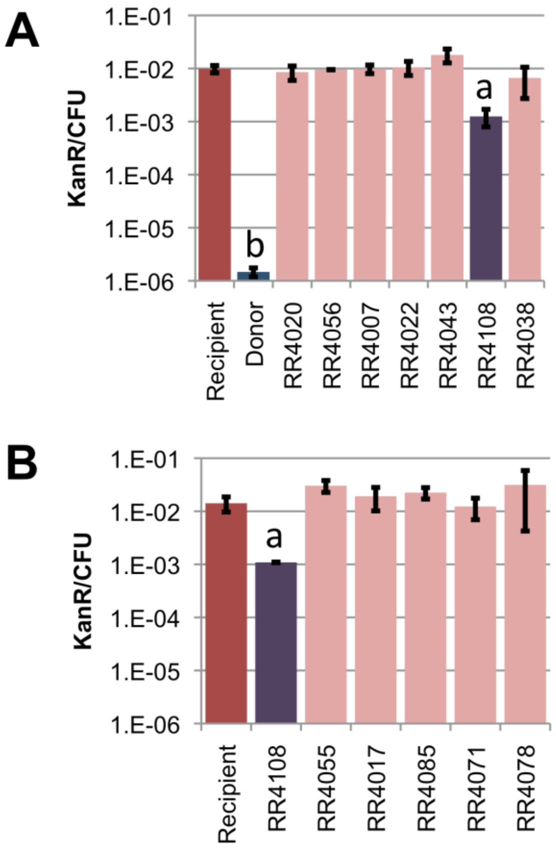

Figure S5 Quantitative retesting of transformability in recombinant clones. Values Kan^{R}/CFU transformation frequencies for MIV starvation cultures and represent the mean of triplicate experiments and error bars show ± standard deviation. Letters indicate significant differences from the Rd recipient strain by paired t-test. (**A**) Retest of the seven recombinants that had shown a reduced transformation frequency in the primary 'transformation-during-growth' assay (purple dots in **Figure 10A**). (**B**) Retest of the six recombinants for which donor segments spanned all or part of a known competence genes. Genes spanned by donor segments are as follows: RR4108 spanned *comM*; RR4055 spanned *sxy* and *rec1*; RR4017 spanned part of *pilA*; RR4085 spanned part of *pilB* and all of *pilCD*; RR4071 spanned *comNOPQ* and *radC*; and RR4078 spanned *ligA*. Known competence-regulated genes not spanned by donor segments were: *comABCDEF*, *comE1*, *pilF2*, *rec2*, and the unknowns *HI0659, HI0660, HI1631,* and *HI0365.*
