# Supplementary material for: Extensive Cotransformation of Natural Variation into Chromosomes of Naturally Competent Haemophilus influenzae
Source: G3 (Bethesda). 2014 Feb 25;4(4):717–31. doi: 10.1534/g3.113.009597 (PMC4059242; doi:10.1534/g3.113.009597)
Supplement: Supporting Information [file supp_g3.113.009597_TableS1.pdf]

**Table S1 Primers Used**

| Name | Purpose | Sequence | Rd-start | Rd-stop | NP-start | NP-stop |
|---|---|---|---|---|---|---|
| F_Rd_A.E10 | test tract 106 | **C**GTTTCTTTATTCTTATTTGG**T**GC | 805,155 | 805,178 | *871,522* | *871,545* |
| R_Rd_A.E10 | test tract 106 | GAAAGTACAAATAG**A**GC**G**AA | 805,496 | 805,477 | *871,863* | *871,844* |
| F_NP_A.E10 | test tract 106 | **T**GTTTCTTTATTCTTATTTGG**A**GC | 805,155 | 805,178 | *871,522* | *871,545* |
| R_NP_A.E10 | test tract 106 | GAAAGTACAAATAG**C**GC**A**AA | 805,496 | 805,477 | *871,863* | *871,844* |
| F_Rd_B.E10 | test tract 106 | AAG**C**CG**C**AAAGAATTACAAGA | 831,046 | 831,066 | *896,435* | *896,455* |
| R_Rd_B.E10 | test tract 106 | TGG**C**GCAGTTTC**A**TCATA | 831,517 | 831,500 | *896,906* | *896,889* |
| F_NP_B.E10 | test tract 106 | AAG**T**CG**T**AAAGAATTACAAGA | 831,046 | 831,066 | *896,435* | *896,455* |
| R_NP_B.E10 | test tract 106 | TGG**T**GCAGTTTC**G**TCATA | 831,517 | 831,500 | *896,906* | *896,889* |
| F_Rd_comM | test tract 143 | GGCGGAA**GTG**TTCGCTGCCT | 1,181,122 | 1,181,141 | *1,214,974* | *1,214,993* |
| F_NP_comM | test tract 143 | GGCGGAA**TCC**TTCGCTGCCT | 1,181,122 | 1,181,141 | *1,214,974* | *1,214,993* |
| R-comM | test tract 143 | GCAGGGCGTAAACCGTGGCAT | 1,186,049 | 1,186,030 | *1,219,901* | *1,219,881* |
| F-murE-Rd | test tract 143 | ACGC**G**CAATGGTGGAAGGCTAC | 1,199,339 | 1,199,360 | *1,233,187* | *1,233,208* |
| F-murE-NP | test tract 143 | ACGC**A**CAATGGTGGAAGGCTAC | 1,199,339 | 1,199,360 | *1,233,187* | *1,233,208* |
| R-murE | test tract 143 | AACAAGCGCAAGCGCACCCT | 1,201,362 | 1,201,343 | *1,235,210* | *1,235,191* |
| F-xylFlank | test tract 143 | AGTCTGCGTGGACATCATCGGA | 1,173,109 | 1,173,130 | *1,210,948* | *1,210,969* |
| R-xylFlank | test tract 143 | CCAGCAAGCGTCGCGTGGTTA | 1,178,735 | 1,178,715 | *1,212,587* | *1,212,567* |
| F-xylG-Rd | test tract 143 | TGCCGTGTTTCTTCCGATCTTCAGG | 1,175,579 | 1,175,603 | *0* | *0* |
| R-xylG-Rd | test tract 143 | ACAGCAGGTGCAATTAGATGCCG | 1,176,249 | 1,176,227 | *0* | *0* |
| *comM*_F | *comMΔ::spc* | GCGGTAAGTTTTTCGGGTAA | 1,182,091 | 1,182,111 | NA | NA |
| *comM*_R | *comMΔ::spc* | CCACACCAAGTTCCCCAAAA | 1,185,779 | 1,185,799 | NA | NA |
| *comM*_*Eco*RI_F | *pSU20-comM* | G**GAATTC**CCTGAAGATACGGGCATTG | 1,182,399 | 1,182,418 | NA | NA |
| *comM*_*Eco*RI_R | *pSU20-comM* | G**GAATTC**GGAGGAAAATAATGACATCAAAT | 1,185,284 | 1,185,307 | NA | NA |

For test primers, bold underlined bases indicate variants between the recipient and donor genomes (RR722 and RR3131). Genome coordinates of primer sequences are provided for both genomes. For cloning primers, bold underlined GAATTC indicates the *Eco*RI restriction site.
