# Supplementary material for: Extensive Cotransformation of Natural Variation into Chromosomes of Naturally Competent Haemophilus influenzae
Source: G3 (Bethesda). 2014 Feb 25;4(4):717–31. doi: 10.1534/g3.113.009597 (PMC4059242; doi:10.1534/g3.113.009597)
Supplement: Supporting Information [file supp_g3.113.009597_FileS1.pdf]

File S1

Supplementary Methods

DNA sequencing, data processing, and short-read alignments: DNA samples were submitted to the Michael Smith Genome Sciences Centre (Vancouver, BC, Canada) for multiplexed Illumina library construction, and the resulting libraries were sequenced in pools of 20-24 per lane on an Illumina HiSeq instrument, yielding ~30 million paired-end reads of 50 nt per end from each lane. Data was provided by the sequencing centre as unmapped BAM files, divided into 91 files based on the samples' multiplexing barcodes.

Datasets were first purged of read pairs failing quality checks. Paired reads that failed the Illumina chastity filter were removed with samtools v0.1.18 (samtools view -F 0x200) (LI *et al.* 2009). The resulting BAM files were then converted to FastQ files using samtools view and the Unix awk utility. The utility sortPairedReads (https://github.com/tanghaibao/trimReads) was used on the resulting FastQs with default settings to set aside read pairs contaminated with Illumina adapter sequences and/or strings of low-quality bases. (The FastA file used for specifying the adaptor sequences included reverse complements.)

The filtered FastQs were then aligned to both donor and recipient reference sequences (Rd KW20 and 86-028NP, GenBank accessions L42023.1 and CP000057.2, respectively) with the BWA short-read aligner (LI and DURBIN 2010)(aln settings: -Y -l 20 -n 8 -o 3 -e 3; sampe used defaults). Mapped sorted BAM files were produced using samtools (view | sort | rmdup). Finally, the GATK IndelRealigner v1.4 was used with default settings to multiply align reads that mapped with short indels and thus minimize alignment artifacts at and near these positions (DEPRISTO *et al.* 2011; MCKENNA *et al.* 2010). Read quality and alignment statistics were collected with FastQC (http://www.bioinformatics.babraham.ac.uk/projects/fastqc/) and samtools flagstat. Read depths and intersects with specific genomic intervals were determined with BedTools v2.16 (see below)(QUINLAN and HALL 2010).

Whole-genome alignment: Alignment of the donor and recipient reference sequences used progressiveMauve v2.3.1 (DARLING *et al.* 2010) with default settings. The resulting XMFA alignments were parsed with a custom R script to generate 'lift-over' tables, which cross-referenced the genome coordinates between the two reference sequences. Because Mauve whole-genome alignment gave slightly different results depending on the order the reference sequences were entered, lift-overs using both reference orders were calculated.

Genotype calling—SNVs: Single-nucleotide variants (SNVs) and short indels (<10 bp) were identified using samtools (samtools mpileup -Eug) with all 91 BAM alignments simultaneously, followed by genotype calling and variant filtering (bcftools view -vg). The two resulting BCF files contained a line for each genomic position where ≥1 sample had a non-reference (alternate) genotype. Because samtools/bcftools were designed to work with diploid genomes, 'heterozygous' or 'mixed' genotypes were called when aligned reads supported both the reference and alternate alleles. Variant call data against both references was deposited as BCFs

Manual inspection of BAM and BCF files used the Integrative Genomics Viewer (IGV) v2.1.17 (THORVALDSDOTTIR *et al.* 2013) to evaluate each filtering step, to validate recombination breakpoints, and to investigate 'mixed' recombination tracts (consisting of contiguous runs of "heterozygous" donor-specific variants). To independently derive allele frequencies at each position, a perl script (https://github.com/riverlee/pileup2base) was used to count bases directly from the pileup output of individual samples.

The two BCF files (corresponding to variant calls against each reference) were decompressed (bcftools view) and parsed (awk) to simplify the output for analysis in R, retaining for each line: the genomic coordinate, the reference allele, the alternate allele(s), and the genotype called for each of the 91 samples (REF for 0/0 reference calls, ALT for 1/1 alternate calls, and MIX for 'heterozygous' 0/1 calls). At the four known MAP7-specific variants in donor strain RR3131 (MELL *et al.* 2011), the reference alleles, alternate alleles, and genotype calls were manually corrected, so that they would correctly identify these donor-specific alleles and escape the stringent filtering described below. To identify a set of 'gold-standard' SNV positions that reliably distinguished donor from recipient, a set of filters were applied to the two genotype files (one corresponding to each reference sequence), as follows and summarized in **TABLE 3**:

(1) The position had a consistent non-zero 'lift-over' to the other genome, as determined by whole-genome alignment. This eliminated many variant calls due to ambiguous alignments around indels and at repetitive sequences. It also eliminated short indel variants, which were handled separately (see below).

(2) Control reads from the donor and recipient strains unambiguously distinguished donor from recipient alleles in the expected fashion, *i.e.* (a) donor reads supported the alternate allele when aligned to the recipient but supported the reference when aligned to the donor, and also (b) recipient reads supported the alternate allele when aligned to the donor but supported the reference when aligned to the recipient. This step recoded the genotypes as 'recipient', 'donor', or 'mixed', and it eliminated variants arising due to differences between the parental strain genomes and their respective references, as well as those with

‘mixed’ genotype calls in the parental controls. This filter also excluded ‘novel’ alleles differing from either parental control, so potentially excluded true mutations that arose in the recombinant clones (see below).

(3) The total fraction of ‘mixed’ genotypes across all 91 sequences was less than 5% (*i.e.* no more than 4 clones had a mixed genotype at a particular position). This filter eliminated error-prone positions resulting from systematic sequencing and alignment errors (particularly for repetitive sequences).

(4) Finally, the ‘gold-standard’ set of variants was reduced to those that passed all three filters, not only for the individual genotype file, but also whose corresponding lift-over position passed all three filters in the reciprocal genotype file.

Genotype calling—SVs: To classify structural variants (SVs), we used a three-step approach, summarized in **Table 4**. First, large indels, inversions, and other rearrangements that distinguish the donor and recipient genomes were identified from the .backbone and “Export gaps…” outputs of progressiveMauve, and the coordinates of each SV were collected for each reference genome. The genomic coordinates of unaligned DNA segments were determined from their flanking aligned breakpoints, and the total set of SV intervals were converted into BED files. Second, the ability of short-read paired-end sequencing to identify these SVs relied on the fact that reads should map to SV breakpoints without gaps or clipping when they are being aligned to the correct genome, but none should map when aligned to the incorrect genome. From each alignment, ungapped unclipped read alignments were extracted at each breakpoint, requiring 5 flanking bases on either side by applying awk and intersectBed. Read depth statistics at each SV breakpoint were extracted using coverageBed. Third, a “gold-standard” set of SVs was determined as those whose breakpoints had high depth when recipient reads were aligned to the recipient genome but low depth against the donor genome and *vice versa*, as determined by Fisher’s exact test (*p-value* < 0.001 per marker). For larger indels (>250 bp) read depth statistics were also extracted from each alignment to confirm the presence/absence of accessory loci. The genotype of each SV for each recombinant was then determined with two Fisher’s exact tests, one against each reference, yielding four potential genotypes: recipient, donor, both, or neither. As with SNVs, SV markers for which >4 samples were both or neither were excluded, since the genotyping method was unreliable at these markers.

Identifying and defining donor segments and breakpoint intervals: Since donor DNAs are known to transform the *H. influenzae* chromosome as relatively long ssDNA molecules, ‘donor segments’ were defined as contiguous runs of gold-standard donor-specific SNVs (including those positions with mixed donor/recipient alleles). Donor segments were called from each genotype file (corresponding to each reference sequence). Breakpoint intervals were initially defined by the coordinate of each donor segments’ outermost donor-specific variants and their nearest adjacent recipient-specific variants. Cross-validation of donor segments in each of the two sets then required that all four breakpoint-defining coordinates in one reference lifted over uniquely to coordinates defining a segment in the other. This cross-validation eliminated most of the putative donor segments with a length of only 1, especially those with mixed genotypes, since many of these arose due to alignment artifacts, rather than representing true transformation events.

Because the “gold-standard” set of SNVs initially excluded indels and other SVs, as well as artifact-prone variants near these and in repetitive DNA, the breakpoint intervals were further refined by interpolation of the SV genotypes. Transforming SVs found within these donor segments were identified as described above and validated by inspection in IGV. Because some DNA samples were mixtures of more than one clone (or the clone was otherwise a mixture of genotypes), contiguous runs of mixed donor/recipient SNVs were deemed ‘mixed’ donor segments. Singleton positions with a mixed genotype (either within or outside donor segments) were manually examined in IGV, and the genotypes were manually adjusted when the donor-specific allele frequency was >90% or <10% (from “mixed” to “donor” or “recipient”, respectively).

References in Supplementary Methods

Darling, A. E., B. Mau and N. T. Perna, 2010 progressiveMauve: multiple genome alignment with gene gain, loss and rearrangement. PLoS One **5:** e11147.

DePristo, M. A., E. Banks, R. Poplin, K. V. Garimella, J. R. Maguire *et al.*, 2011 A framework for variation discovery and genotyping using next-generation DNA sequencing data. Nat Genet **43:** 491-498.

Li, H., and R. Durbin, 2010 Fast and accurate long read alignment with Burrows-Wheeler transform. Bioinformatics.

Li, H., B. Handsaker, A. Wysoker, T. Fennell, J. Ruan *et al.*, 2009 The Sequence Alignment/Map format and SAMtools. Bioinformatics **25:** 2078-2079.

McKenna, A., M. Hanna, E. Banks, A. Sivachenko, K. Cibulskis *et al.*, 2010 The Genome Analysis Toolkit: a MapReduce framework for analyzing next-generation DNA sequencing data. Genome Res **20:** 1297-1303.

Mell, J. C., S. Shumilina, I. M. Hall and R. J. Redfield, 2011 Transformation of natural genetic variation into Haemophilus influenzae genomes. PLoS Pathog **7:** e1002151.

Quinlan, A. R., and I. M. Hall, 2010 BEDTools: a flexible suite of utilities for comparing genomic features. Bioinformatics **26:** 841-842.

Thorvaldsdottir, H., J. T. Robinson and J. P. Mesirov, 2013 Integrative Genomics Viewer (IGV): high-performance genomics data visualization and exploration. Brief Bioinform **14:** 178-192.
